# Dynamic Prediction in Mixture Cure Models: A Model‐Based Landmarking Approach

**DOI:** 10.1002/sim.70774

**Published:** 2026-10-18

**Authors:** Marta Cipriani, Marco Alfò, Mirko Signorelli

**Affiliations:** ^1^ Department of Statistical Sciences Sapienza University of Rome Rome Italy; ^2^ School of Mathematics and Statistics University College Dublin Dublin Ireland; ^3^ Mathematical Institute Leiden University Leiden the Netherlands

**Keywords:** dynamic prediction, mixed‐effects models, mixture cure models, model‐based landmarking

## Abstract

Mixture cure models are widely used in survival analysis when a portion of patients is considered cured and is no longer at risk for the event of interest. In clinical settings, dynamic survival prediction is particularly important to refine prognosis by incorporating patient information updated over time. Landmarking methods have emerged as a flexible approach for this purpose, as they allow to summarize longitudinal covariates up to a given landmark time and use these summaries in subsequent prediction. For mixture cure models, the only landmarking strategy available in the literature relies on summarizing the dynamics of observed longitudinal markers through the last observation carried forward (LOCF) method. However, LOCF discards most of the longitudinal information, does not correct for measurement error, and may rely on outdated values if observation times are far apart. To overcome these limitations, we propose a sequential approach that integrates model‐based landmarking within a mixture cure model. Initially, longitudinal covariates are modeled using (generalized) linear mixed models, from which individual‐specific summaries are predicted. The predicted summaries are then incorporated as covariates into a Cox proportional hazards cure model. We investigated the performance of the proposed approach under different cure fractions, sample sizes, and longitudinal data structures through an extensive simulation study. The results show that the model‐based strategy provides more refined predictions compared to LOCF, even when the model is misspecified in favor of the LOCF approach. Finally, we illustrate our method using a real‐world dataset on renal transplant patients.

## 1 Introduction

Dynamic prediction models are crucial tools in clinical research, as they leverage baseline and follow‐up data to provide updated survival predictions as new information becomes available. This ability to adapt predictions over time is particularly valuable in personalized medicine, where therapeutic strategies can be tailored to the evolving profile of each patient.

Several approaches have been developed to implement dynamic prediction. The time‐dependent Cox model [1], for example, incorporates time‐varying covariates directly into the hazard function, offering a natural extension of the classical proportional hazards framework. Joint models provide a more integrated solution by simultaneously modeling the longitudinal trajectory of covariates and the survival process, typically by considering shared (or correlated) individual‐specific random effects that link the two submodels [2, 3]. An alternative and flexible approach is landmarking, which builds upon prediction models at pre‐specified time points, known as landmark times, using only individuals still at risk at those times [4]. In landmarking, repeated measures of time‐dependent covariates observed up to the landmark time are first summarized, either through simple synthetic functions or more refined statistical models, and then used as predictors in survival models alongside baseline covariates.

The way in which longitudinal information is summarized clearly plays a central role in determining the performance of these approaches. Landmarking methods can be broadly categorized into *traditional* and *model‐based* approaches. Traditional methods [4] are based on simple strategies, such as those based on the last observation carried forward (LOCF) or the average of previous measurements. In contrast, model‐based approaches employ more sophisticated statistical tools to capture the underlying structure of longitudinal data. These include linear mixed‐effects models (LMM) and multivariate functional principal component analysis (MFPCA), both of which allow rich summaries of the time‐varying covariates. Such model‐based summaries have been used in combination with several prediction models, including MFPCA with Cox models [5, 6], MFPCA with random survival forests [7], LMMs with random survival forests [8], and LMMs with penalized Cox models [9, 10].

As interest in prediction methods increases, it has also become evident that the assumption of homogeneity in survival models is often violated. In such cases, cure models offer a valuable framework by explicitly accounting for a subgroup of people who are effectively *cured* and not at risk of experiencing the event of interest, even with long‐term follow‐up [11]. The two most established approaches in this framework are the mixture cure model (MCM) [12, 13] and the promotion time cure model (PTCM) [14]. The MCM explicitly separates cured and susceptible individuals, allowing for independent modeling of the two components, which makes it highly interpretable and flexible. PTCM, on the other hand, avoids explicit classification and models the cure fraction through a hazard‐based approach, offering a smoother representation of the transition between risk and cure, but typically requires stronger assumptions on the baseline hazard structure. Given its interpretability and flexibility for modeling, in the following we will focus on the MCM only.

Extending dynamic prediction to settings with a cure fraction has recently attracted growing interest. Most of the existing proposals—with the exception of Shi and Yin [15], who combined landmarking and MCM using the LOCF strategy—have been developed in the context of PTCMs [16, 17, 18, 19, 20, 21]. The approach of Shi and Yin [15] represents, to our knowledge, the only attempt to implement landmarking within an MCM framework. It relies on a simple LOCF approach, which may fail to capture the full longitudinal evolution of covariates, especially when the last observation and the landmark time are far apart, and it is unable to correct for the measurement error that is often present when biomarkers are used as covariates. In addition, the adopted definition of a time‐dependent cure probability raises conceptual and methodological concerns that will be discussed in more detail in Section 2.3. In this work, we propose an alternative model‐based landmarking method for dynamic prediction in MCMs to address these issues.

The remainder of this paper is organized as follows. Section 2 introduces the data structure, the notation, and the basics on MCM and landmarking. Section 3 presents the proposed methodology: the model‐based landmarking approach and its four main steps (Section 3.1), followed by the metrics used to evaluate predictive performance (Section 3.2). Section 4 reports the design and results of a large‐scale simulation study, and Section 5 illustrates the application to a real‐world case study. Finally, Section 6 concludes with a discussion of the main findings, limitations, and directions for future research.

## 2 Notation and Background

### 2.1 Data Structure and Notation

We consider a dataset with m individuals. For subject i=1,…,m, let ${\tilde{t}}_{i}$ and $c_{i}$ denote the true event and censoring times, respectively, and let $t_{i}=\min\left({\tilde{t}}_{i},c_{i}\right)$ be the observed time. The event indicator is denoted by $\delta_{i}=I\left({\tilde{t}}_{i}\leq c_{i}\right)$, where I(a) is the indicator function for condition a.

In addition, we assume that we have observed p covariates (indexed by l=1,…,p) of two types:

- $p_{Y}$ *longitudinal covariates* $\;Y_{i}$. We allow for a completely unbalanced study design in continuous time, whereby the l‐th longitudinal covariate for subject i is measured at $n_{\mathrm{il}}$ time points 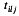, $l=1,\ldots ,p_{Y}$, 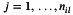. To keep the notation simple, however, in what follows we assume a common measurement schedule for each subject and write $t_{\mathrm{ij}}$, $j=1,\ldots ,n_{i}$. We denote by $n=\sum_{i=1}^{m}n_{i}$ the total number of observations. The vector with the $n_{i}$ observations of the l‐th longitudinal covariate for subject i is

$$y_{\mathrm{il}}={\left(y_{\mathrm{il}}\left(t_{i1}\right),\ldots ,y_{\mathrm{il}}\left(t_{{\text{in}}_{i}}\right)\right)}^{\prime }.$$

- $p-p_{Y}$ *time‐fixed covariates*, which we partition into $x_{i}$ and $z_{i}$, representing two vectors of (possibly overlapping) baseline covariates that will be linked to different components of the MCM.

### 2.2 Mixture Cure Models Without Longitudinal Covariates

MCMs assume that the population is divided into *cured* (G=0) and *uncured* (G=1) individuals. Cured individuals will never experience the event of interest (e.g., relapse after treatment), whereas uncured individuals may eventually do so. However, the cure status G is not always observable in practice, and for each individual the model provides an estimate for the probability of being uncured.

This probability, that is, the *incidence* component, is described by a binary response model, typically a logistic regression:

(1) $$P\left(G_{i}=1|x_{i}\right)=\pi \left(x_{i}\right)=\frac{\exp\left(\alpha_{0}+\alpha^{\prime }x_{i}\right)}{1+\exp\left(\alpha_{0}+\alpha^{\prime }x_{i}\right)}.$$

For uncured individuals, the survival distribution conditional on covariates $z_{i}$ is referred to as the *latency* component, and it is commonly specified through a Cox proportional hazards (PH) model:

(2) $$S_{u}\left(t|z_{i}\right)=\exp\left[-H_{0}\left(t\right)\exp\left(\;\beta^{\prime }z_{i}\right)\right],$$

where the subscript u refers to the *uncured* portion of the population and $H_{0}\left(t\right)$ is the baseline cumulative hazard. The overall survival function is then

(3) $$S\left(t|x_{i},z_{i}\right)=\left[1-\pi \left(x_{i}\right)\right]+\pi \left(x_{i}\right)S_{u}\left(t|z_{i}\right).$$

Since this model depends on the latent cure status G, the set of parameters is usually estimated by maximum likelihood using an Expectation–Maximization (EM) algorithm [22, 23].

Although mixture cure models are not limited to a PH formulation, see Amico and Van Keilegom [11] for a review, the Cox PH cure model is a common choice.

### 2.3 Landmarking in Mixture Cure Models: The LOCF Approach

We consider a mixture cure setting in which landmarking is used to incorporate longitudinal information observed up to a pre‐specified time $t_{\ell_{i}}$, referred to as the *landmark time*, to predict survival at a future horizon $t_{\omega_{i}}$. The notation $t_{\ell_{i}}$ allows for subject‐specific landmark times, as in settings with a natural landmark (e.g., the time of transplantation for patients on a waiting list). In the present work, however, we focus on the simpler case of a common landmark defined in patient time, so that $t_{\ell_{i}}=t_{\ell }$ for all individuals.

The only previous attempt to combine landmarking with MCMs is due to Shi and Yin [15], who proposed a class of landmark cure rate models that incorporate both a cure fraction and time‐dependent covariates for dynamic prediction. In their approach, the probability of being uncured is specified through a logistic function depending on time‐dependent covariates,

(4) $$P\left(G_{i}=1|y_{i}\left(t\right)\right)=\pi \left(y\left(t\right)\right)=\frac{\exp\left(\;\alpha_{0}+\alpha^{\prime }y_{i}\left(t\right)\right)}{1+\exp\left(\;\alpha_{0}+\alpha^{\prime }y_{i}\left(t\right)\right)},$$

where the incidence is defined as the limit

$$P\left(G_{i}=1\right)=\lim_{t\to \infty }\pi \left(y_{i}\left(t\right)\right).$$

At a given landmark time $t_{\ell }$, they propose fitting a standard mixture cure model with covariates $y_{i}\left(t_{\ell }\right)$ if available. Whenever the longitudinal covariates are not measured at landmark time, they suggest using the last measurement available, according to a LOCF strategy.

While innovative, this construction raises two conceptual issues:

- The cure probability is linked to the limiting value y(∞) of time‐dependent covariates. This implies that the cure status of an individual depends on future covariate values, even in scenarios where the subject could already have experienced the event before those covariates are realized (e.g., death before transplantation in their motivating example). This specification is not coherent with the idea that the prediction at $t_{\ell }$ should be based only on the information available up to that time.
- By construction, Shi and Yin [15] write the cure probability as in (4). However, from a probabilistic point of view, it can be shown that

$$\lim_{t\to \infty }P\left(G=1|y\left(t\right)\right)\neq P\left(G=1|y\left(t_{\ell }\right)\right)$$

and the error associated to such an approximation cannot be controlled. Therefore, their specification may lead to theoretical inconsistency.

To address these limitations, we assume that cure status is determined solely by baseline covariates, while longitudinal covariates influence only the survival distribution of uncured individuals. Specifically, in our case, Equations (2) and (3) can be rewritten, respectively, as

(5) $$S_{u}\left(t|z_{i},y_{i}\right)=\exp\left[-H_{0}\left(t\right)\exp\left(\beta^{\prime }z_{i}+\psi^{\prime }y_{i}\left(t\right)\right)\right],$$

and

(6) $$S\left(t|x_{i},z_{i},y_{i}\right)=\left[1-\pi \left(x_{i}\right)\right]+\pi \left(x_{i}\right)S_{u}\left(t|z_{i},y_{i}\left(t\right)\right).$$

This ensures that the prediction at the landmark time relies exclusively on the information available up to that point. Furthermore, the cure status is constant over time, and the corresponding probability is defined in a coherent and identifiable way. In fact, it is often clinically reasonable to regard the propensity to cure as fixed at baseline, reflecting inherent patient‐ or disease‐specific characteristics rather than post‐baseline evolution.

## 3 Methods

### 3.1 Model‐Based Landmarking in Cure Models

We propose a model‐based landmarking method for MCMs in which a statistical model is used to describe the dynamics of longitudinal covariates up to the landmark time. The summaries derived from these models are then combined with the baseline information for prediction. The procedure follows four main steps, inspired by Signorelli et al. [10]:

1. Model the longitudinal covariates via (G)LMMs;
2. Summarize the longitudinal histories through appropriate (individual‐specific) quantities, for example, predicted random effects;
3. Estimate the MCM using an EM algorithm;
4. Generate post‐landmark predictions.

We describe these steps in detail below.

#### 3.1.1 Step 1: Modeling the Longitudinal Covariates

The dynamics in the longitudinal covariates up to the landmark time are described by using either a linear mixed model (LMM) [24] or a generalized linear mixed model (GLMM) [25].

Specifically, we fit a separate (generalized) LMM for each longitudinal covariate. For non‐Gaussian responses, we follow the formulation of Achana et al. [26], which linearizes the model regardless of the response and provides an alternative implementation of penalized quasi‐likelihood in the spirit of Breslow and Clayton [27]. The model is fitted using only measurements collected up to the landmark time $t_{\ell_{i}}$ and restricted to individuals still at risk at $t_{\ell_{i}}$. Let $ℛ\left(t_{\ell }\right)=\left\{i\in \left\{1,\ldots ,m\right\}:t_{i}\geq t_{\ell }\right\}$ denote the risk set at the landmark time and let $m_{\ell }=\;|ℛ\left(t_{\ell }\right)|$ be the corresponding size.

For each at‐risk subject i, the l‐th longitudinal covariate at time $t_{\mathrm{ij}}\leq t_{\ell_{i}}$ is modeled, conditionally on the subject‐specific random effects $b_{\mathrm{il}}$, through a generalized linear mixed model. On the scale of the link function, the conditional mean satisfies

(7) $$g\left(E\left[\;Y_{\mathrm{il}}\left(t_{\mathrm{ij}}\right)|b_{\mathrm{il}}\;\right]\right)=w_{\mathrm{il}}{\left(t_{\mathrm{ij}}\right)}^{\prime }\gamma_{l}+v_{\mathrm{il}}{\left(t_{\mathrm{ij}}\right)}^{\prime }b_{\mathrm{il}},\;t_{\mathrm{ij}}\leq t_{\ell_{i}},$$

where $Y_{\mathrm{il}}\left(t_{\mathrm{ij}}\right)|b_{\mathrm{il}}$ follows a distribution in the exponential family. Here

- g(⋅) is a differentiable monotonic link function;
- $w_{\mathrm{il}}\left(t_{\mathrm{ij}}\right)\in {\mathbb{R}}^{p_{W}}$ is the vector of fixed‐effects covariates for subject i and covariate l at time $t_{\mathrm{ij}}$; it typically contains baseline covariates (elements of $x_{i}$ and $z_{i}$) and polynomials in t;
- $\gamma_{l}\in {\mathbb{R}}^{p_{W}}$ is the vector of fixed effects for the l‐th covariate;
- $v_{\mathrm{il}}\left(t_{\mathrm{ij}}\right)\in {\mathbb{R}}^{p_{V}}$ is a subvector of $w_{\mathrm{il}}\left(t_{\mathrm{ij}}\right)$, typically including a constant term (an individual‐specific intercept) and polynomials in t to describe individual‐specific dynamics, as in latent growth models [28];
- $b_{\mathrm{il}}∼\mathrm{MVN}\left(0,\Sigma_{b_{l}}\right)$ is the vector of individual‐specific random effects, capturing heterogeneity across subjects.

When the response is Gaussian, the link is the identity and the model reduces to the linear mixed model

(8) $$Y_{\mathrm{il}}\left(t_{\mathrm{ij}}\right)=w_{\mathrm{il}}{\left(t_{\mathrm{ij}}\right)}^{\prime }\gamma_{l}+v_{\mathrm{il}}{\left(t_{\mathrm{ij}}\right)}^{\prime }b_{\mathrm{il}}+\epsilon_{\mathrm{il}}\left(t_{\mathrm{ij}}\right),\;t_{\mathrm{ij}}\leq t_{\ell_{i}},$$

with $\epsilon_{\mathrm{il}}\left(t_{\mathrm{ij}}\right)∼N\left(0,\sigma_{l}^{2}\right)$ independent of $b_{\mathrm{il}}$, for $i=1,\ldots ,m_{\ell }$, $j=1,\ldots ,n_{i}\left(t_{\ell_{i}}\right)$, $l=1,\ldots ,p_{Y}$, where $n_{i}\left(t_{\ell_{i}}\right)$ denotes the number of measurements available for subject i up to $t_{\ell_{i}}$. Stacking these $n_{i}\left(t_{\ell_{i}}\right)$ observations of subject i, we write $W_{\mathrm{il}}$ ($n_{i}\left(t_{\ell_{i}}\right)\times p_{W}$) and $V_{\mathrm{il}}$ ($n_{i}\left(t_{\ell_{i}}\right)\times p_{V}$) for the corresponding design matrices, whose j‐th rows are $w_{\mathrm{il}}{\left(t_{\mathrm{ij}}\right)}^{\prime }$ and $v_{\mathrm{il}}{\left(t_{\mathrm{ij}}\right)}^{\prime }$, and $\epsilon_{\mathrm{il}}∼\mathrm{MVN}\left(0,\Sigma_{\varepsilon_{\mathrm{il}}}\right)$ with $\Sigma_{\epsilon_{\mathrm{il}}}=\sigma_{l}^{2}I_{n_{i}\left(t_{\ell_{i}}\right)}$.

#### 3.1.2 Step 2: Summarizing Longitudinal Covariates at the Landmark Time

At the landmark time, each patient's longitudinal history is summarized through a derived quantity, say $\hat{s}\left(y_{\mathrm{il}}\left(t_{\ell_{i}}\right)\right)$ for the l‐th longitudinal covariate and the i‐th individual. This summary can be either:

- The predicted random effects ${\hat{b}}_{\mathrm{il}}$, or
- The predicted value at the landmark time, ${\hat{y}}_{\mathrm{il}}\left(t_{\ell_{i}}\right)$,

obtained from the (G)LMM fitted to the l‐th longitudinal covariate up to $t_{\ell_{i}}$ in Step 1. Both these summaries provide a concise representation of individual‐specific heterogeneity in observed dynamics and can be treated as time‐constant predictors in the cure model. In particular, the predicted random effects provide a low‐dimensional representation of subject‐specific trajectory features (e.g., individual level and slope), conditional on the observed data and the estimated model parameters. The predicted value at the landmark time is based on a combination of fixed effects and random effects. The fixed effects in the longitudinal model describe average population trends, while the random effects capture individual deviations from these average patterns; therefore, the two components together summarize the dynamic information that is considered most relevant for personalized prediction at the landmark time.

For the LMM case, the predicted random effects ${\hat{b}}_{\mathrm{il}}$ are calculated as follows:

(9) $${\hat{b}}_{\mathrm{il}}=E\;\left(b_{\mathrm{il}}|\;y_{\mathrm{il}}\right)={\hat{\Sigma }}_{b_{l}}\;V_{\mathrm{il}}^{\prime }{\left[\;V_{\mathrm{il}}{\hat{\Sigma }}_{b_{l}}\;V_{\mathrm{il}}^{\prime }+{\hat{\Sigma }}_{\epsilon_{\mathrm{il}}}\right]}^{-1}\left(\;y_{\mathrm{il}}-W_{\mathrm{il}}\;{\hat{\gamma }}_{l}\right)$$

that is, the estimator derived using the conditional distribution of $b_{\mathrm{il}}|y_{\mathrm{il}}$ obtained exploiting the joint Gaussianity of $\left(y_{\mathrm{il}},b_{\mathrm{il}}\right)$. The variance components ${\hat{\Sigma }}_{b_{l}}$ and ${\hat{\Sigma }}_{\epsilon_{\mathrm{il}}}$ are estimated from the fitted LMM via restricted maximum likelihood (REML).

When the longitudinal outcome does not satisfy the Gaussian assumption, we fit a generalized linear mixed model. The marginal density of $y_{\mathrm{il}}$ is given by

(10) $$f_{Y}\left(y_{\mathrm{il}}\right)=\int f_{Y}\left(y_{\mathrm{il}}|b_{\mathrm{il}}\right)f_{B}\left(b_{\mathrm{il}}\right)\;db_{\mathrm{il}},$$

where $f_{Y}\left(y_{\mathrm{il}}|b_{\mathrm{il}}\right)$ is the conditional density specified by the regression model as a function of covariates $W_{\mathrm{il}}$ and random effects $b_{\mathrm{il}}$, and $f_{B}\left(b_{\mathrm{il}}\right)$ is the prior density for the random effects.

In both LMMs and GLMMs, if the correlation between observed covariates $W_{\mathrm{il}}$ and unobserved heterogeneity $b_{\mathrm{il}}$ is an issue, it can be addressed through a Mundlak‐type reparameterization [29], adding cluster‐level means of time‐varying covariates to the fixed effects part of the model.

Since the integral in the marginal density (10) does not have a closed form, a numerical approximation is required. Following Wolfinger and O'Connell [30], we adopt a *penalized quasi‐likelihood* (PQL) approach, which relies on a first‐order Taylor expansion to linearize the model on the link scale, yielding the *linearized response* $y_{\mathrm{il}}^{*}$. As in the LMM case, the predicted random effects ${\hat{b}}_{\mathrm{il}}$ are then obtained as empirical Bayes estimates from the conditional distribution of $b_{\mathrm{il}}|y_{\mathrm{il}}^{*}$.

The predicted value of a longitudinal covariate at the landmark time is obtained as

$${\hat{y}}_{\mathrm{il}}\left(t_{\ell }\right)=g^{-1}\left(w_{\mathrm{il}}{\left(t_{\ell }\right)}^{\prime }{\hat{\gamma }}_{l}+v_{\mathrm{il}}{\left(t_{\ell }\right)}^{\prime }{\hat{b}}_{\mathrm{il}}\right).$$

#### 3.1.3 Step 3: Estimation of the Cure Model Parameters

Once summaries for observed time‐dependent covariates have been obtained, we consider the set of subjects still at risk at the landmark time and estimate a mixture cure model. We then incorporate both baseline covariates $\left(x_{i},z_{i}\right)$ and the summaries $\hat{s}\left(y_{i}\left(t_{\ell_{i}}\right)\right)$ derived in Step 2 into the cure model. All likelihood contributions are implicitly conditional on survival up to $t_{\ell }$.

Specifically, while the incidence component is as in (1), the latency in (2) becomes

$$S_{u}\left(t|z_{i},y_{i}\right)=\exp\left[-H_{0}\left(t\right)\exp\left(\;\beta^{\prime }z_{i}+\psi^{\prime }\hat{s}\left(y_{i}\left(t_{\ell_{i}}\right)\right)\right)\right].$$

where t≥0 represents the time elapsed since the landmark time $t_{\ell }$.

If we denote by θ the *complete* set of parameters of interest, the complete‐data likelihood function for the Cox PH cure model, based on the $m_{\ell }$ individuals in $ℛ\left(t_{\ell }\right)$, can be written as

(11) $$\begin{array}{ll} {ℒ}_{c}\left(\;\theta \;\right) & =\prod_{i=1}^{m_{\ell }}{\left[\pi \left(x_{i}\right)f_{u}\left(T_{i}|z_{i},y_{i}\right)\right]}^{\delta_{i}G_{i}}{\left[\pi \left(x_{i}\right)S_{u}\left(T_{i}|z_{i},y_{i}\right)\right]}^{\left(1-\delta_{i}\right)G_{i}}\\ & \;\;{\left[1-\pi \left(x_{i}\right)\right]}^{\left(1-\delta_{i}\right)\left(1-G_{i}\right)} \end{array}$$

In the E‐step of the EM algorithm [31], we calculate the posterior expectation of the latent indicator $G_{i}$. For individual i∈(1,…m) at iteration r, this is given by

(12) $$q_{i}^{\left(r\right)}=\delta_{i}+\left(1-\delta_{i}\right)\frac{\pi^{\left(r-1\right)}\left(x_{i}\right)S_{u}^{\left(r-1\right)}\left(T_{i}|\;z_{i},{\hat{y}}_{i}\right)}{\left[1-\pi^{\left(r-1\right)}\left(x_{i}\right)\right]+\pi^{\left(r-1\right)}\left(x_{i}\right)S_{u}^{\left(r-1\right)}\left(T_{i}|z_{i},{\hat{y}}_{i}\right)}$$

In the M‐step, we maximize the expected complete‐data log‐likelihood conditional on survival up to $t_{\ell }$, replacing $G_{i}$ by its posterior expectation $q_{i}^{\left(r\right)}$. The objective function decomposes into incidence and latency components,

$$Q\left(\;\theta \;|\;\theta^{\left(r\right)}\right)=Q_{1}\left(\;\theta \;|\;\theta^{\left(r\right)}\right)+Q_{2}\left(\;\theta \;|\;\theta^{\left(r\right)}\right),$$

with summations taken over $i=1,\ldots ,m_{\ell }$. Additional details on this M‐step decomposition are provided in the Supporting Information materials.

Maximization with respect to $H_{0}$ at iteration r leads to the following modified Breslow estimator [32] of the baseline cumulative hazard function

(13) $${\hat{H}}_{0}^{\left(r\right)}\left(t\right)=\sum_{i=1}^{m_{\ell }}\frac{I\left(t_{i}<t\right)\delta_{i}}{\sum_{i\in ℛ\left(t_{\ell }\right)}q_{i}^{\left(r\right)}\exp\left(\beta^{\left(r-1\right)\prime }\;z_{i}+\psi^{\left(r-1\right)\prime }\hat{s}\left(y_{i}\left(t_{\ell_{i}}\right)\right)\right)}$$

where the numerator is the number of events up to time t.

Standard errors for the estimated parameters are obtained from the observed information matrix using the method of Louis [33]. This provides asymptotic standard errors for both incidence and latency parameters, allowing for inference while maintaining consistency with the EM‐based estimation framework.

#### 3.1.4 Step 4: Post‐Landmark Prediction

Once the regression parameters have been estimated, we can derive the conditional cure and survival probabilities by plugging in the estimated values:

(14) $$\hat{\pi }\left(x_{i}|t_{\ell_{i}}\right)=\frac{\exp\left(\;{\hat{\alpha }}_{0}+\hat{\alpha^{\prime }}x_{i}\right)}{1+\exp\left({\hat{\alpha }}_{0}+{\hat{\alpha }}^{\prime }x_{i}\right)},$$

(15) $${\hat{S}}_{u}\left(t_{\omega_{i}},z_{i},{\hat{y}}_{i}|t_{\ell_{i}}\right)=\exp\left[-{\hat{H}}_{0}\left(t_{\omega_{i}}\right),\exp\left(\;\beta^{\prime }z_{i}+\psi^{\prime }\hat{s}\left(y_{i}\left(t_{\ell_{i}}\right)\right)\right)\right].$$

Predictions can be obtained for both subjects in the estimation set and for out‐of‐sample individuals, since they rely on the parameter estimates obtained in Step 3. This naturally raises the question of how to evaluate the quality of such predictions. The next section describes the accuracy measures that we suggest employing for this purpose.

### 3.2 Metrics for Predictive Performance Evaluation

In the MCM framework, predictive performance is typically assessed separately [34] for the *incidence* and the *latency* component. The prediction targets are, respectively, the cure status $G_{i}$ and the residual survival of susceptible subjects.

For the incidence component, since the true cure status $G_{i}$ is only partially observed, we rely on weighted versions of standard classification metrics, where each subject is weighted by its estimated posterior probability of being uncured ${\hat{q}}_{i}$. Discrimination between cured and uncured subjects is quantified by the *weighted AUC* [35],

$${\hat{\mathrm{AUC}}}_{\mathrm{inc}}=\frac{\sum_{i\neq j}I\left({\hat{\eta }}_{i}^{\left(I\right)}>{\hat{\eta }}_{j}^{\left(I\right)}\right)\;{\hat{q}}_{i}\;\left(1-{\hat{q}}_{j}\right)}{\sum_{i\neq j}{\hat{q}}_{i}\;\left(1-{\hat{q}}_{j}\right)},$$

where ${\hat{\eta }}_{i}^{\left(I\right)}={\hat{\alpha }}_{0}+{\hat{\alpha }}^{\prime }x_{i}$ is the incidence linear predictor and $\hat{q}$ is the posterior expectation in (12) obtained at the convergence of the EM algorithm. The corresponding prediction error is measured by the *weighted Brier score* [36],

$${\hat{\mathrm{BS}}}_{\mathrm{inc}}=\frac{1}{m}\sum_{i=1}^{m}{\hat{u}}_{i}\;{\left({\hat{q}}_{i}-{\tilde{G}}_{i}\right)}^{2},$$

where ${\tilde{G}}_{i}=1$ if the subject i experienced the event, ${\tilde{G}}_{i}=0$ if censored beyond the largest observed event time, and ${\hat{u}}_{i}$ are the inverse probability of censoring weights (IPCW), that is, the inverse of the Kaplan–Meier estimate of the probability of remaining uncensored up to the observed time.

For the latency component, prediction performance is evaluated using standard survival measures modified for censoring. Discrimination at time t is assessed by the *time‐dependent AUC* [37],

$$\hat{\mathrm{AUC}}{\left(t\right)}_{\mathrm{lat}}=\frac{\sum_{i\neq j}I\left({\hat{\eta }}_{i}^{\left(L\right)}>{\hat{\eta }}_{j}^{\left(L\right)}\right)\;\delta_{i}\left(t\right)\;\left(1-\delta_{j}\left(t\right)\right)\;{\hat{u}}_{i}\left(t\right)\;{\hat{u}}_{j}\left(t\right)}{\sum_{i\neq j}\delta_{i}\left(t\right)\;\left(1-\delta_{j}\left(t\right)\right)\;{\hat{u}}_{i}\left(t\right)\;{\hat{u}}_{j}\left(t\right)},$$

where ${\hat{\eta }}_{i}^{\left(L\right)}=\beta^{\prime }z_{i}+\psi^{\prime }\hat{s}\left(y_{i}\left(t_{\ell_{i}}\right)\right)$ is the latency linear predictor and $\delta_{i}\left(t\right)$ indicates whether subject i experienced the event before t. Prediction error for the survival probability at time t is measured by the *time‐dependent Brier score* [38],

$$\hat{\mathrm{BS}}{\left(t\right)}_{\mathrm{lat}}=\frac{1}{m}\sum_{i=1}^{m}{\hat{u}}_{i}\left(t\right)\;{\left(I\left(t_{i}>t\right)-{\hat{S}}_{i}\left(t\right)\right)}^{2},$$

where ${\hat{S}}_{i}\left(t\right)$ is the predicted survival probability for subject i and ${\hat{u}}_{i}\left(t\right)$ are the corresponding IPCW weights. Finally, the overall concordance between predicted risk and observed event times can be summarized by the *concordance index (C‐index)* [39, 40],

$$\hat{C}=\frac{\sum_{i\neq j}I\left({\hat{\eta }}_{i}^{\left(L\right)}>{\hat{\eta }}_{j}^{\left(L\right)}\right)\;D_{i,j}}{\sum_{i\neq j}D_{i,j}},$$

with $D_{i,j}=I\left[t_{i}<t_{j},\delta_{i}=1\right]+I\left[t_{i}=t_{j},\delta_{i}=1,\delta_{j}=0\right]$.

## 4 Simulation Study

### 4.1 Simulation Design

We conducted a simulation study to evaluate the performance of the proposed model‐based approach for dynamic prediction in mixture cure models, comparing the random‐effects (*Model‐based RE*) and predicted‐value (*Model‐based PV*) implementations to a LOCF strategy. The study considered different sample sizes, cure fractions, number of repeated measurements, and data‐generating mechanisms.

For each setting, 1000 datasets including four time‐invariant and four time‐varying covariates were generated. For subject i and longitudinal covariate l=1,…,4, repeated measurements were generated according to the model

(16) $$Y_{\mathrm{il}}\left(t\right)=\eta_{\mathrm{il}}\left(t\right)+\varepsilon_{\mathrm{il}}\left(t\right),$$

where the latent trajectory is defined as

(17) $$\eta_{\mathrm{il}}\left(t\right)=b_{0,\mathrm{il}}+b_{1,\mathrm{il}}t,$$

with subject‐specific random intercepts and slopes

$$b_{0,\mathrm{il}}∼N\left(0,1\right),\;b_{1,\mathrm{il}}∼N\left(0,0.7\right),$$

and independent measurement errors $\varepsilon_{\mathrm{il}}\left(t\right)∼N\left(0,1\right)$. Measurement times $t_{\mathrm{ij}}$ were independently sampled from a $\text{Unif}\left(0,t_{\ell }\right)$ distribution and sorted, with landmark time fixed at $t_{\ell }=3$. The same measurement times were shared by all four longitudinal covariates within each subject, mirroring a setting in which all biomarkers are recorded together at each visit.

Two longitudinal designs were considered: a *balanced* design with $M_{i}=10$ measurements per subject, and an *unbalanced* design obtained by randomly deleting some of the observation times so that $M_{i}\in \left[5,10\right]$. Under the unbalanced design, the LOCF strategy, applied after the amputation step, relied on the last observed measurement after deletion, which could occur well before the landmark. More precisely, for each subject i and longitudinal covariate l, the quantity $Y_{\mathrm{il}}\left(t_{\ell }\right)$ used in Equations (5) and (6) was defined as the last available observed value prior to (or at) $t_{\ell }$, that is,

$$y_{\mathrm{il}}\left(t_{\ell }\right)=y_{\mathrm{il}}\left(t_{{\mathrm{ij}}^{*}}\right),\;t_{{\mathrm{ij}}^{*}}=\max\left\{t_{\mathrm{ij}}\leq t_{\ell }\right\}.$$

From the landmark onwards, all subjects were considered at risk, with event times simulated from a Weibull distribution conditional on the time‐varying covariates and regression coefficients β=(1,0,−1,0). Independent censoring times were drawn from an exponential distribution starting at the landmark. Under this specification, the average overall censoring rate ranged between approximately 38% and 55% across the considered cure‐fraction scenarios.

To assess the robustness of the proposed model under different data‐generating assumptions, three mechanisms were considered for the latency component, corresponding to different ways of linking the longitudinal process to the survival model:

- *LOCF‐based scenario*. Survival times are generated using the *observed* longitudinal process in (16), so that the hazard at the landmark depends directly on the noisy measurements. To be more specific, we used a Weibull baseline hazard with relative risk defined by the following specification

$$h\left(t|y_{\mathrm{il}}\left(t_{\ell }\right),b_{\mathrm{il}}\right)=h_{0}\left(t\right)\exp\left(\;\beta^{\prime }y_{i}\left(t_{\ell }\right)\right)$$

ii *Current value (CV) scenario*. Survival times are generated assuming the hazard depends on the *true underlying value* of the longitudinal process at time t, denoted as $\eta_{\mathrm{il}}\left(t\right)=E\left[\;Y_{\mathrm{il}}\left(t\right)|b_{\mathrm{il}}\right]$. That is, the hazard is defined as

$$h\left(t|y_{\mathrm{il}}\left(t_{\ell }\right),b_{\mathrm{il}}\right)=h_{0}\left(t\right)\exp\left(\;\beta^{\prime }\eta_{i}\left(t_{\ell }\right)\right)$$

iii *Random effects (RE) scenario*. Survival times are generated using the subject‐specific random effects underlying the longitudinal process, as in (17). Here, the association is only with the random deviations (e.g., intercepts and slopes) of the longitudinal submodel.

For each of the 1000 simulated datasets, predictions were obtained on an external validation dataset, generated independently but under the same data‐generating mechanism as the corresponding training dataset.

Time‐invariant covariates $x_{i}={\left(x_{i1},\ldots ,x_{i4}\right)}^{\prime }$ were generated from a multivariate normal distribution $N_{4}\left(0,I\right)$. The cure status was generated from a logistic regression model for the probability of *not* being cured,

$$\mathrm{Pr}\left(G_{i}=1|x_{i}\right)={\text{logit}}^{-1}\;\left(\alpha_{0}+\alpha^{\prime }x_{i}\right),$$

where $\alpha ={\left(-1,0,1,0\right)}^{\prime }$. Different cure fractions were obtained by varying the intercept $\alpha_{0}$. Specifically, an intercept value $\alpha_{0}=2$ yielded, on average, a cure fraction of 20%, whereas $\alpha_{0}=0.65$ yielded 40%.

Each combination of cure fraction and longitudinal design (balanced or unbalanced) was tested at two sample sizes (m=300,1000), resulting in four basic scenarios replicated twice.

An overview of all simulation scenarios is provided in Table 1. All code and materials to reproduce the simulation study are available in a dedicated GitHub repository at https://github.com/martacip/cure_prediction.

**TABLE 1:** Simulation scenarios with scenario numbers. Each scenario is defined by cure fraction, longitudinal design, and generating mechanism.

Scenario	Cure fraction	Longitudinal design	Generating mechanism
1	20%	Balanced ($M_{i}=10$)	LOCF‐based mechanism
2	20%	Unbalanced ($M_{i}\in \left[5,10\right]$)	LOCF‐based mechanism
3	40%	Balanced ($M_{i}=10$)	LOCF‐based mechanism
4	40%	Unbalanced ($M_{i}\in \left[5,10\right]$)	LOCF‐based mechanism
5	20%	Balanced ($M_{i}=10$)	CV‐based mechanism
6	20%	Unbalanced ($M_{i}\in \left[5,10\right]$)	CV‐based mechanism
7	40%	Balanced ($M_{i}=10$)	CV‐based mechanism
8	40%	Unbalanced ($M_{i}\in \left[5,10\right]$)	CV‐based mechanism
9	20%	Balanced ($M_{i}=10$)	RE‐based mechanism
10	20%	Unbalanced ($M_{i}\in \left[5,10\right]$)	RE‐based mechanism
11	40%	Balanced ($M_{i}=10$)	RE‐based mechanism
12	40%	Unbalanced ($M_{i}\in \left[5,10\right]$)	RE‐based mechanism

### 4.2 Simulation Results

The results for the incidence component are reported in Table 2. Since both LOCF and the model‐based approaches rely on the same specification for the cure model, their predictive performance in terms of incidence is essentially indistinguishable across scenarios. Small fluctuations are observed, but these appear to be random and unrelated to the sample size or design and do not seem to indicate any systematic advantage of either method.

**TABLE 2:** Incidence results for each scenario (mean [SD]) by sample size.

Scenario	LOCF (AUC; Brier)	Model‐based PV (AUC; Brier)	Model‐based RE (AUC; Brier)
Sample size n=300			
1	0.75 [0.04]; 0.15 [0.01]	0.75 [0.04]; 0.15 [0.01]	0.75 [0.04]; 0.15 [0.01]
2	0.75 [0.04]; 0.15 [0.01]	0.75 [0.04]; 0.15 [0.01]	0.75 [0.04]; 0.15 [0.01]
3	0.77 [0.03]; 0.19 [0.01]	0.77 [0.03]; 0.19 [0.01]	0.77 [0.03]; 0.19 [0.01]
4	0.77 [0.03]; 0.19 [0.01]	0.77 [0.03]; 0.19 [0.01]	0.77 [0.03]; 0.19 [0.01]
5	0.75 [0.04]; 0.15 [0.01]	0.75 [0.04]; 0.15 [0.01]	0.75 [0.04]; 0.15 [0.01]
6	0.75 [0.04]; 0.15 [0.01]	0.75 [0.04]; 0.15 [0.01]	0.75 [0.04]; 0.15 [0.01]
7	0.77 [0.03]; 0.19 [0.01]	0.77 [0.03]; 0.19 [0.01]	0.77 [0.03]; 0.19 [0.01]
8	0.77 [0.03]; 0.19 [0.01]	0.77 [0.03]; 0.19 [0.01]	0.77 [0.03]; 0.19 [0.01]
9	0.75 [0.04]; 0.15 [0.01]	0.75 [0.04]; 0.15 [0.01]	0.75 [0.04]; 0.15 [0.01]
10	0.75 [0.04]; 0.15 [0.01]	0.75 [0.04]; 0.15 [0.01]	0.75 [0.04]; 0.14 [0.01]
11	0.77 [0.03]; 0.19 [0.01]	0.77 [0.03]; 0.19 [0.01]	0.77 [0.03]; 0.19 [0.01]
12	0.77 [0.03]; 0.19 [0.01]	0.77 [0.03]; 0.19 [0.01]	0.77 [0.03]; 0.19 [0.01]
Sample size n=1000			
1	0.76 [0.02]; 0.14 [0.01]	0.76 [0.02]; 0.14 [0.01]	0.76 [0.02]; 0.14 [0.01]
2	0.76 [0.02]; 0.15 [0.01]	0.76 [0.02]; 0.15 [0.01]	0.76 [0.02]; 0.14 [0.01]
3	0.78 [0.01]; 0.19 [0.01]	0.78 [0.01]; 0.19 [0.01]	0.78 [0.01]; 0.19 [0.01]
4	0.77 [0.01]; 0.19 [0.01]	0.77 [0.01]; 0.19 [0.01]	0.78 [0.01]; 0.19 [0.01]
5	0.76 [0.02]; 0.14 [0.01]	0.76 [0.02]; 0.14 [0.01]	0.76 [0.02]; 0.14 [0.01]
6	0.76 [0.02]; 0.14 [0.01]	0.76 [0.02]; 0.14 [0.01]	0.76 [0.02]; 0.14 [0.01]
7	0.78 [0.01]; 0.19 [0.01]	0.78 [0.01]; 0.19 [0.01]	0.78 [0.01]; 0.19 [0.01]
8	0.77 [0.01]; 0.19 [0.01]	0.77 [0.01]; 0.19 [0.01]	0.77 [0.01]; 0.19 [0.01]
9	0.76 [0.02]; 0.14 [0.01]	0.76 [0.02]; 0.14 [0.01]	0.76 [0.02]; 0.14 [0.01]
10	0.76 [0.02]; 0.15 [0.01]	0.76 [0.02]; 0.14 [0.01]	0.76 [0.02]; 0.14 [0.01]
11	0.78 [0.01]; 0.19 [0.01]	0.78 [0.01]; 0.19 [0.01]	0.78 [0.01]; 0.19 [0.01]
12	0.78 [0.01]; 0.19 [0.01]	0.78 [0.01]; 0.19 [0.01]	0.78 [0.01]; 0.19 [0.01]

*Note:* Scenario numbers refer to Table 1. Results are reported for LOCF and model‐based approaches (both with predicted values and random effects), with weighted AUC and weighted Brier score.

Figures 1, 2, 3 show the time‐dependent AUC for latency prediction under the LOCF, CV, and RE mechanisms, respectively.

**FIGURE 1:**
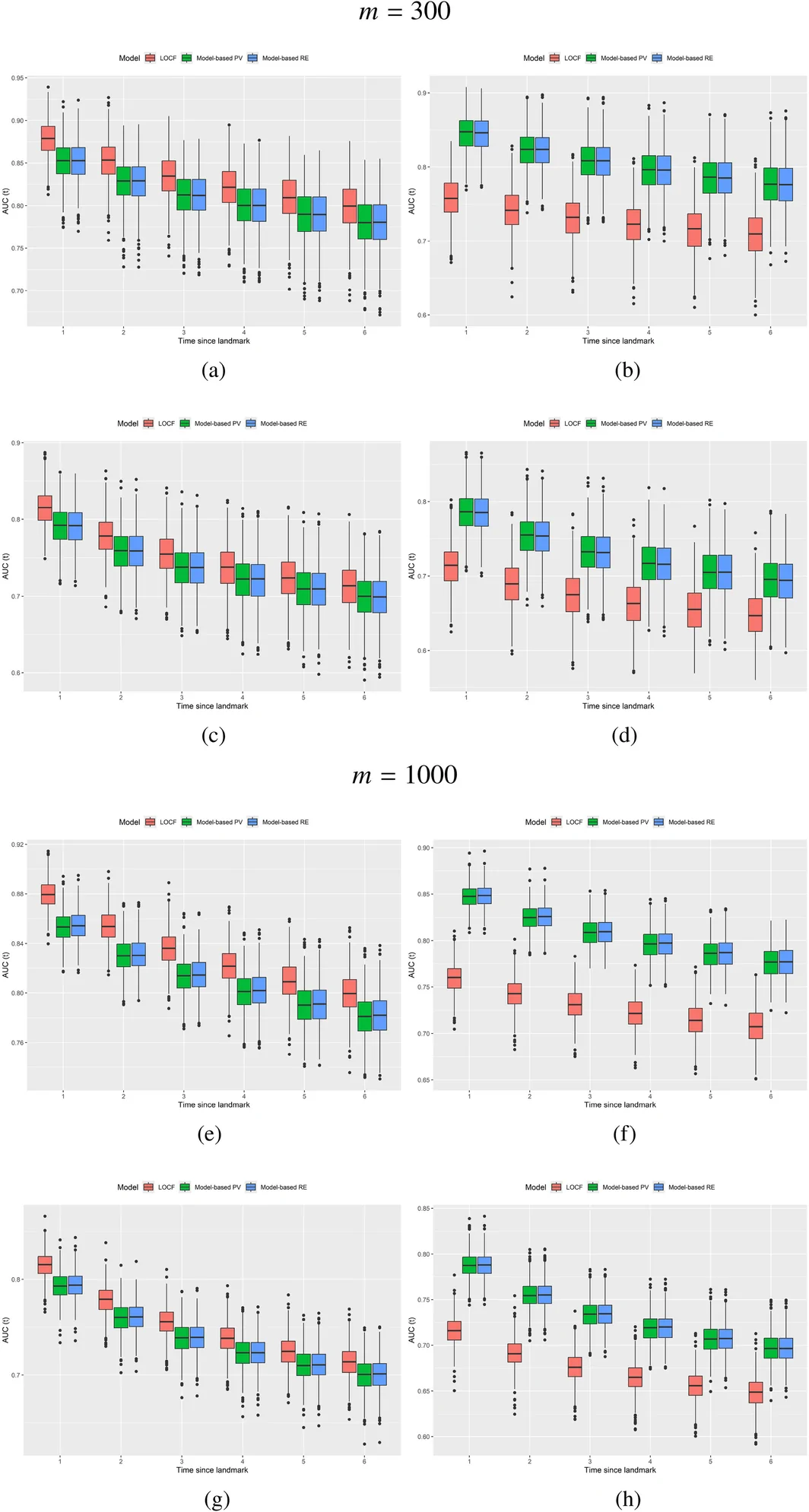
Time‐dependent AUC for scenarios under LOCF‐based mechanism. (a) Scenario 1%–20% cured; *M* balanced; (b) scenario 2%–20% cured; *M* unbalanced; (c) scenario 3%–40% cured; *M* balanced; (d) scenario 4%–40% cured; *M* unbalanced; (e) scenario 1%–20% cured; *M* balanced; (f) scenario 2%–20% cured; *M* unbalanced; (g) scenario 3%–40% cured; *M* balanced; (h) scenario 4%–40% cured; *M* unbalanced.

**FIGURE 2:**
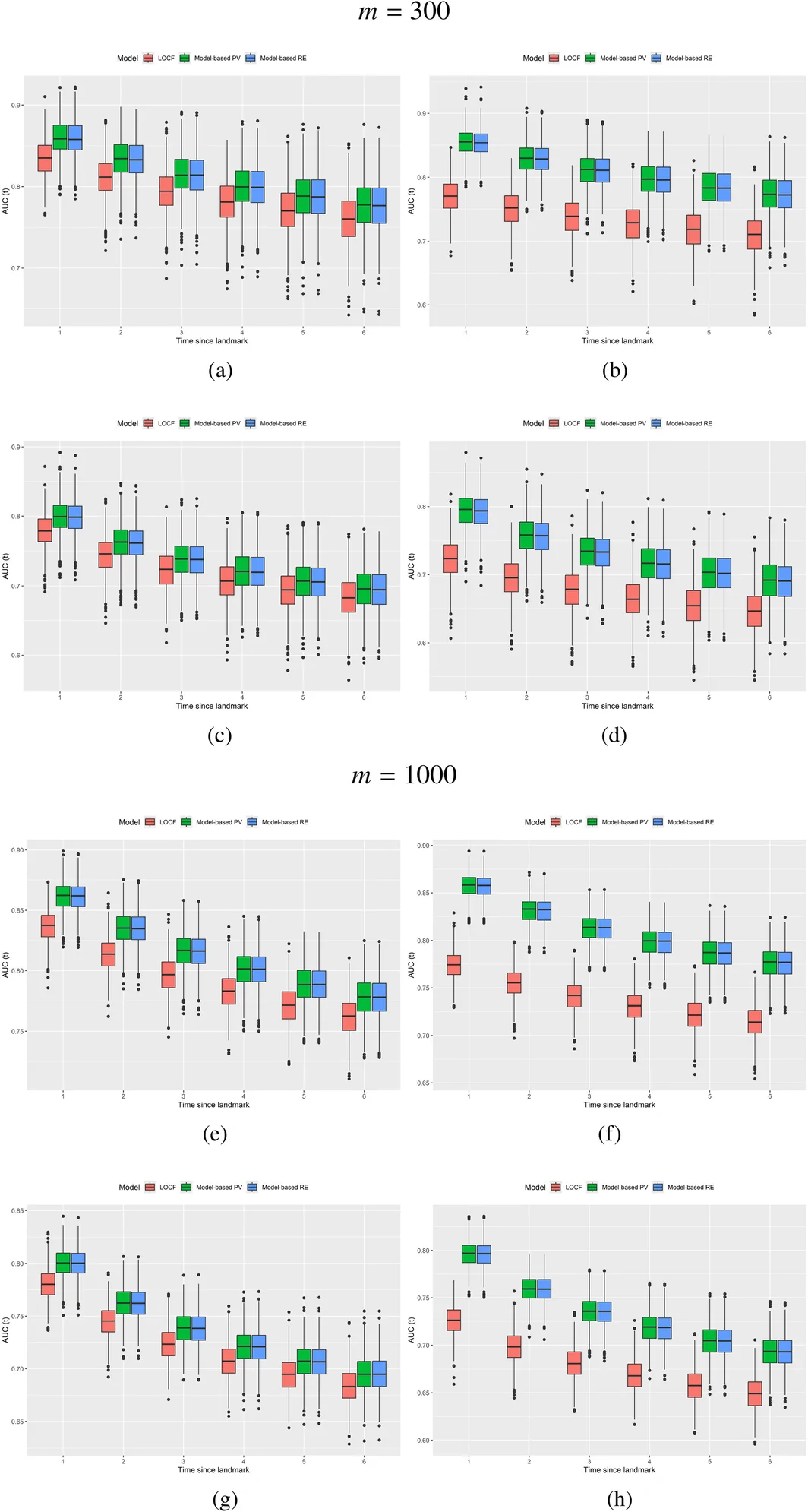
Time‐dependent AUC for scenarios under CV‐based mechanism. (a) Scenario 5%–20% cured; *M* balanced; (b) scenario 6%–20% cured; *M* unbalanced; (c) scenario 7%–40% cured; *M* balanced; (d) scenario 8%–40% cured; *M* unbalanced; (e) scenario 5%–20% cured; *M* balanced; (f) scenario 6%–20% cured; *M* unbalanced; (g) scenario 7%–40% cured; *M* balanced; (h) scenario 8%–40% cured; *M* unbalanced.

**FIGURE 3:**
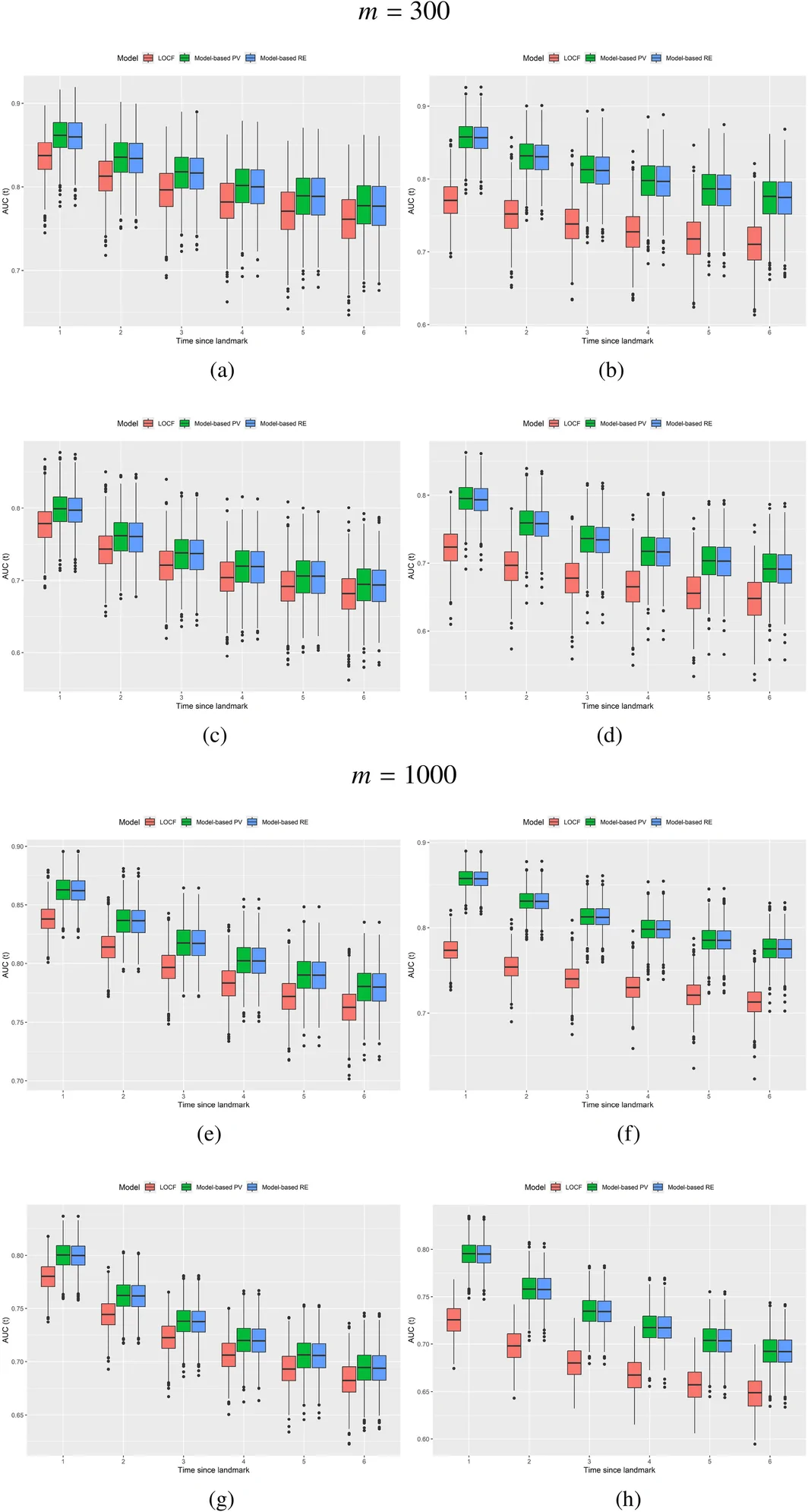
Time‐dependent AUC for scenarios under RE‐based mechanism. (a) Scenario 9%–20% cured; *M* balanced; (b) scenario 10%–20% cured; *M* unbalanced; (c) scenario 11%–40% cured; *M* balanced; (d) scenario 12%–40% cured; *M* unbalanced; (e) scenario 9%–20% cured; *M* balanced; (f) scenario 10%–20% cured; *M* unbalanced; (g) scenario 11%–40% cured; *M* balanced; (h) scenario 12%–40% cured; *M* unbalanced.

Under the LOCF setting (Figure 1) and a balanced longitudinal study, the LOCF approach outperformed the *model‐based* strategy. This finding is expected because, in this specific design, LOCF coincides with the true data‐generating mechanism. However, when the longitudinal design was unbalanced, the proposed *model‐based* approach outperformed the LOCF approach even though survival times were generated from the LOCF mechanism. This is explained by the fact that, due to irregular deletion, the LOCF estimation relied on measurements that could be substantially earlier than the landmark, effectively making the fitted LOCF model itself misspecified. This led to a marked loss of predictive performance, in line with the well‐documented limitations of LOCF‐based approaches under irregular observation patterns [41, 42].

When moving to a CV setting (Figure 2) or to a RE setting (Figure 3), the proposed *model‐based* method consistently outperformed LOCF across all scenarios. This reflects the ability of the model‐based formulation to correctly capture individual trajectories.

The model‐based approach relying on predicted values at the landmark (PV) exhibited performance almost indistinguishable from the model‐based relying on the random effects inclusion (RE) across all data‐generating mechanisms. This result is not surprising, as both approaches are derived from the same mixed‐effects model and exploit the same subject‐specific information available up to the landmark. In particular, the PV approach evaluates the fitted trajectory at the landmark time using the empirical Bayes estimates of the random effects, whereas the RE approach includes those random effects explicitly as covariates in the survival model. Since both strategies rely on the same underlying longitudinal structure, their predictive performance is nearly identical in all scenarios.

To further explore potential differences, we also included in the Supporting Information material additional simulations in which the process generating the latent longitudinal trajectory is based also on fixed effects that are not explicitly included as predictors in the cure survival model. Specifically, for subject i and longitudinal covariate l=1,…,4, we generated the latent process as

(18) $$\eta_{\mathrm{il}}\left(t\right)=b_{0,\mathrm{il}}+b_{1,\mathrm{il}}t+\gamma_{l}^{\prime }w_{\mathrm{il}}\left(t\right)$$

with $\;\gamma_{l}$ simulated from a uniform distribution, for example, $\gamma_{1l}∼\text{Uniform}\left(1,2\right)$. This setup allows assessing the robustness of the RE and PV approaches when part of the longitudinal variation is not explicitly modeled in the survival component. In this setting, the PV approach moderately outperforms the RE approach, while both model‐based strategies clearly outperform the LOCF method.

Importantly, these conclusions across all considered data‐generating mechanisms and longitudinal designs were consistent for both m=300 and m=1000, indicating that the comparative performance of the methods was not driven by the sample size. Additional performance metrics, reported in Figures S2–S4 of the Supporting Information material, confirmed the patterns observed looking at the time‐dependent AUC.

## 5 Case Study: Renal Transplant Data

### 5.1 Data Description and Statistical Modeling

We illustrate the proposed methodology using the renal dataset from the joineRML R package [43]. This dataset comprises 407 patients with chronic kidney disease who received a primary renal transplant from either a deceased or living donor at the University Hospital of the Catholic University of Leuven (Belgium) between January 1983 and August 2000. Chronic kidney disease is characterized by progressive loss of renal function, typically assessed through repeated measurements of glomerular filtration rate (GFR).

From a clinical perspective, renal transplantation is a potentially curative intervention, as successful transplantation restores kidney functions and long‐term survival can be similar to that of the general population [44, 45]. Accordingly, the Kaplan–Meier survival curve for this dataset (Figure 4) exhibits a plateau, a hallmark of cure scenarios [11]. In addition, such datasets have been widely used in methodological studies on joint modeling and dynamic prediction for renal transplant outcomes [46, 47], providing a rich framework for demonstrating the performance of advanced prediction techniques for graft failure.

**FIGURE 4:**
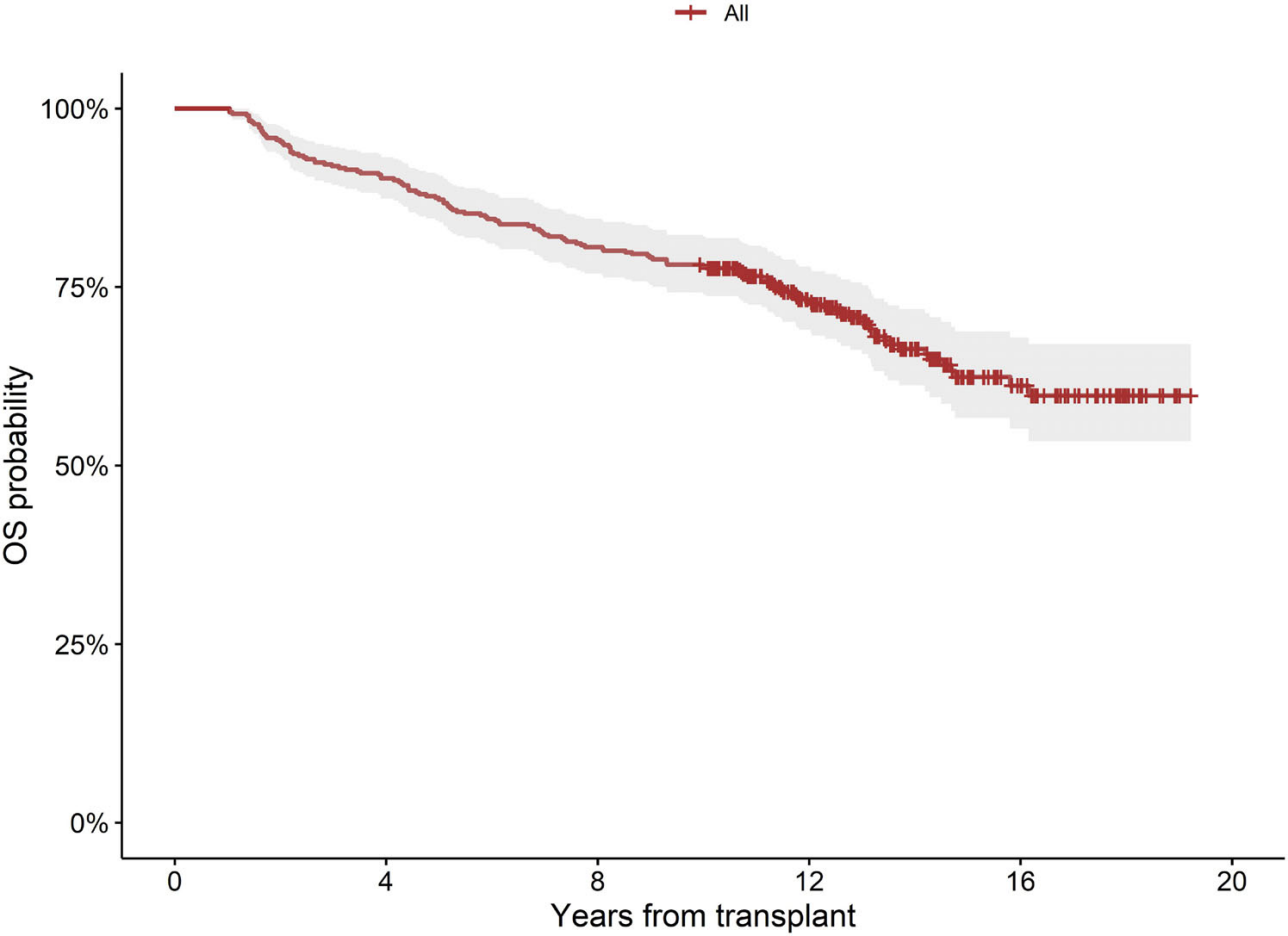
Kaplan–Meier survival curve for renal transplant patients.

For each patient, multiple longitudinal biomarkers were recorded over time, along with a time‐to‐event outcome indicating graft failure. We used baseline covariates, such as age, weight, and sex in the incidence submodel, with age categorized according to quartiles. Besides the marginal effect of age we also considered its interaction with sex. In the latency submodel, only longitudinal biomarkers reflecting kidney function were included, namely hematocrit (% of red blood cells in blood) and GFR (mL/min/1.73 m^2^). For these two biomarkers, we report in Table 3 the average number of measurements per individual at landmark times 1, 1.5, 2, 2.5 and 3 years after transplantation.

**TABLE 3:** Average number of longitudinal measurements per patient at selected landmark times.

Landmark time (years)	Hematocrit	GFR
1.0	54.07	59.25
1.5	61.55	68.44
2.0	67.31	75.96
2.5	72.69	82.39
3.0	77.56	87.93

In the first step of the proposed method, each longitudinal biomarker was modeled using a linear mixed‐effects model. To maintain computational simplicity and interpretability at the individual level, each model included only a random intercept and a random slope for time, allowing patient‐specific deviations in both baseline levels and trajectories over time. This specification mirrors that used in the simulation study, without incorporating any additional model validation strategy.

### 5.2 Results

Based on the findings from our simulation study, where the performance of the model‐based approach using predicted values (PV) was often similar to those obtained using random effects (RE), we focused on comparing the *model‐based RE* with the LOCF strategy. For simplicity and clarity, from this point onward, whenever we refer to the model‐based approach, we specifically mean the model‐based approach using RE. We used repeated cross validation with 4 folds and 10 repetitions to evaluate predicted performance.

It is important to note that the renal transplant dataset represents an atypical scenario: each patient has a very large number of repeated measurements (Table 3), which can make modeling individual trajectories more challenging, while the abundance of data naturally favors LOCF. Despite these data characteristics, two distinct patterns emerged.

First, the model‐based approach shows an increasing relative advantage as the prediction horizon extends further from the fixed landmark time (typically after the first 2 years), as illustrated by the time‐dependent AUC curves in Figure 5. This highlights the benefit of explicitly modeling subject‐specific trajectories rather than relying on the last available observation, which quickly loses relevance as time progresses.

**FIGURE 5:**
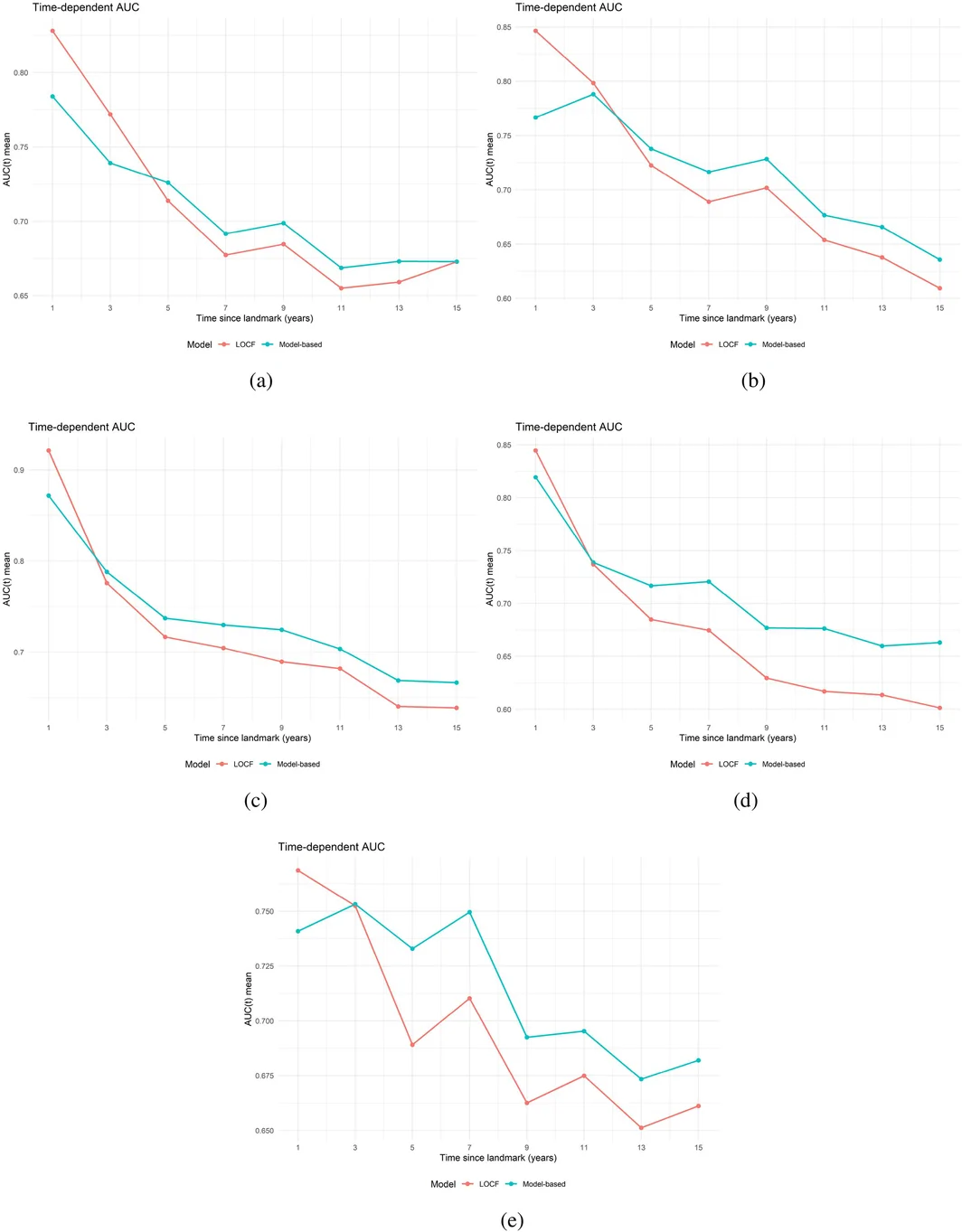
Time‐dependent AUC curves for the model‐based and LOCF approaches at multiple landmark times. (a) Landmark time *t* = 1; (b) landmark time *t* = 1.5; (c) landmark time *t* = 2; (d) landmark time *t* = 2.5; (e) landmark time *t* = 3.

Second, when landmarks are set further from the baseline, the model‐based approach also tends to perform better, since additional longitudinal information is available to inform the mixed‐effects model. We can also notice that later landmarks are associated with fewer events available to estimate survival, leading to greater variability in performance metrics.

Only AUC results are reported here; results based on the time‐dependent Brier score, provided in Figure S6 of the Supporting Information material, showed trends consistent with the AUC but with smaller discrepancies between the two methods and reduced variability. In particular, both the evolution across landmark times and the performance decay when moving further from the landmark time appeared more stable with the Brier score. For clarity, Figure 5 displays AUC curves without standard errors, while plots including standard errors for both the AUC and the Brier score are available in the Supporting Information material (Figures S5 and S7, respectively).

Table 4 reports the C‐index for the latency submodel, providing a global measure of discriminative ability across landmarks. These values confirm the pattern observed with the AUC curves, with the model‐based approach generally outperforming the LOCF‐based one.

**TABLE 4:** Latency metrics (C‐index) at different landmark times (years) for model‐based and LOCF approaches.

Landmark time (years)	Model	C‐index mean	C‐index SD
1	Model‐based	0.66	0.03
1	LOCF	0.66	0.05
1.5	Model‐based	0.68	0.05
1.5	LOCF	0.65	0.05
2	Model‐based	0.69	0.06
2	LOCF	0.67	0.06
2.5	Model‐based	0.68	0.06
2.5	LOCF	0.64	0.07
3	Model‐based	0.69	0.06
3	LOCF	0.67	0.06

For the incidence submodel, performance (Table 5) is broadly comparable between the model‐based and LOCF‐based approaches, consistent with the simulation study, reflecting that baseline covariates largely drive event occurrence predictions.

**TABLE 5:** Incidence metrics (AUC and Brier) at different landmark times for model‐based and LOCF approaches.

Landmark time (years)	Model	AUC mean	AUCSD	Briermean	BrierSD
1	Model‐based	0.76	0.03	0.23	0.03
1	LOCF	0.76	0.03	0.23	0.03
1.5	Model‐based	0.74	0.03	0.23	0.03
1.5	LOCF	0.75	0.03	0.24	0.03
2	Model‐based	0.77	0.03	0.22	0.03
2	LOCF	0.77	0.03	0.23	0.03
2.5	Model‐based	0.78	0.03	0.22	0.03
2.5	LOCF	0.79	0.03	0.22	0.04
3	Model‐based	0.77	0.03	0.21	0.03
3	LOCF	0.78	0.03	0.22	0.03

## 6 Discussion

In this work, we developed a methodology for dynamic prediction in the framework of mixture cure models with longitudinal covariates. Our approach is based on a model‐based landmarking strategy in which we employ a mixed‐effects model to obtain subject‐specific summaries of the longitudinal biomarkers' trajectories. The method makes it possible to include longitudinal covariates into the latency component while maintaining computational tractability, making a step further in the literature on cure models, which so far has mainly focused on static covariates and LOCF‐based landmarking.

The simulation study provided systematic evidence of the advantages of the model‐based approach. Even when the data‐generating mechanism coincided with LOCF, the proposed method performed competitively. Furthermore, once the longitudinal design became unbalanced, the proposed strategy showed clear superiority, reflecting the well‐known limitations of LOCF‐based approaches when measurement times are irregular. Under more realistic data‐generating processes, the model‐based approach consistently outperformed the LOCF‐based one across all sample sizes, cure fractions, and longitudinal designs. These findings highlight the robustness of the proposed method under different data‐generating mechanisms and its ability to leverage longitudinal information more effectively. Importantly, improvements were observed for both moderate and large sample sizes, suggesting that the benefits are not linked to the sample size.

The renal transplant case study further illustrated the practical relevance of our methodology. This dataset presents an atypical setting: patients undergo frequent follow‐up measurements, leading to an abundance of longitudinal data that naturally favors the LOCF‐based approach. Nevertheless, our results demonstrated that the model‐based approach provides increasing predictive gains as the prediction horizon extends further from the landmark. This reflects its ability to model underlying trajectories rather than relying on a single past measurement, which becomes increasingly uninformative over time. At the same time, we observed the inherent trade‐off of landmarking methods: while later landmarks benefit from richer longitudinal histories, they are associated with fewer events, which translates into greater variability of predictive metrics.

In both simulation and real data, a consistent pattern emerged between the incidence and latency components of the cure model. As the incidence component is primarily based on baseline covariates, the prediction performance for this component remained comparable between the two methods. In contrast, for the latency submodel, which directly incorporates longitudinal information, we observed substantial differences, with the model‐based strategy showing clear advantages. This confirms the importance of explicitly modeling individual trajectories when the focus is on residual survival among the susceptible population.

From a methodological perspective, our study emphasizes the value of embedding longitudinal processes into cure models for prediction. While the LOCF‐based approach remains attractive for its simplicity, it is prone to inaccuracy in settings with irregular follow‐up and offers limited flexibility for extrapolating subject‐specific trends. The proposed approach provides a principled alternative that strikes a balance between interpretability, computational feasibility, and predictive accuracy.

An additional advantage of our landmarking strategy is its computational efficiency compared to full joint longitudinal‐survival models. Joint models offer a coherent framework for dynamic prediction by simultaneously capturing the evolution of longitudinal biomarkers and the survival outcome. Our sequential approach can be viewed as a pragmatic approximation: individual‐specific summaries of the longitudinal trajectories are first extracted and then incorporated into the survival component. While this strategy does not fully account for the uncertainty in the longitudinal process as joint models do, it substantially reduces computational complexity. This makes the landmarking approach particularly attractive in settings with large datasets, multiple biomarkers, or frequent measurements, while still capturing the key subject‐specific trajectory information relevant for survival prediction.

Despite its advantages, the proposed methodology also has some limitations. A key aspect of the model‐based strategy is that it requires the specification of an appropriate mixed‐effects model for the longitudinal trajectories in the first step. In practice, selecting the best‐fitting structure (e.g., choice of random effects, covariance structure, or functional form of time) may not be straightforward, especially when data exhibit complex or highly irregular patterns. Misspecification at this stage can propagate to the prediction step, potentially reducing the accuracy of the latency component. In the simulation study, linear mixed‐effects models were adopted for clarity and to ensure a controlled comparison between approaches. However, the proposed framework does not inherently rely on linear trajectory assumptions. In real applications, more flexible specifications may be required. Indeed, in the renal transplant case study, the observed biomarker profiles exhibit marked nonlinear patterns, highlighting that simple linear time trends may be insufficient to capture the underlying dynamics. In such settings, spline‐based formulations or nonlinear time effects can be incorporated within the mixed‐effects modeling step. While these extensions increase modeling accuracy, they also introduce additional challenges in terms of interpretation, computational burden, and risk of overfitting.

A practical question concerns the choice between the two longitudinal summaries introduced in Section 3.1. The RE summary contributes one predictor per random effect (typically an intercept and a slope term), whereas the PV summary contributes a single predictor per covariate; with many biomarkers or richer random‐effects structures, RE thus yields a higher‐dimensional latency model, with heavier computational cost in the EM step and greater exposure to overfitting. This additional flexibility, however, may also be an advantage: by keeping level and slope as distinct predictors, RE can achieve a higher predictive performance than PV whenever these features act on the hazard in different ways. Since both summaries derive from the same mixed model fitted in Step 1, estimating and comparing them is inexpensive, and we recommend this as the default approach: the choice can be guided by the predictive performance measures, or one may conclude—as in our simulations, where the two performed often quite similarly—that they are equivalent in predictive accuracy. When a direct comparison is not desirable, the decision may rest on subject‐matter knowledge of which trajectory features are expected to drive the outcome, or on computational considerations, in which case the parsimony of PV makes it a sensible default. A mild advantage for PV emerged only in the Supporting Information scenario, where part of the longitudinal signal was carried by fixed effects omitted from the survival model.

Future research may extend this framework in several directions. Incorporating multivariate longitudinal processes, for instance, through latent process mixed models, would allow joint modeling of multiple biomarkers that reflect a common biological pathway.

Another potential improvement concerns the estimation of the incidence component across landmark times. In the current implementation, the cure model is estimated separately at each landmark time, implicitly allowing the regression coefficients of the incidence component to vary across landmarks. However, when the same baseline covariates are used at each landmark, one may reasonably assume that the underlying incidence mechanism remains stable over time. In such cases, efficiency could potentially be improved by borrowing information across landmarks, for example by pooling the corresponding estimates of α or by imposing a common incidence model across landmark times.

An intermediate strategy would be to allow the incidence coefficients to vary smoothly as a function of the landmark time, for instance through spline‐based parametrizations or other smoothing approaches. This would provide additional flexibility while preserving stability of the estimates, particularly at later landmarks where the number of events is smaller. Exploring such joint or semi‐parametric formulations represents an interesting direction for future methodological developments.

Finally, we note that in the present work the landmark time was fixed in patient time and assumed common to all individuals. However, in other applications, the landmark time may arise naturally and be subject‐specific, such as the time of transplantation for patients on a waiting list, after which survival prediction is of interest. In such settings, landmarking requires careful methodological adjustments because the length of the follow‐up prior to the landmark differs across subjects. As shown by Gomon et al. [6], this may induce a selection bias in the estimation of both fixed and random effects of the longitudinal model, ultimately degrading predictive performance. Developing strategies, for instance, through appropriate reweighting, to account for individual‐specific landmarks while preserving consistency of dynamic prediction in cure models represents an important direction for future research.

## Funding

This work was supported by Sapienza University of Rome, Progetti di Ricerca Grandi (protocol no. RG124191029C1395) and Progetti per Avvio alla Ricerca (protocol no. AR1251999A136D8A), and by the Italian Ministry of University and Research (MUR) under PRIN2022 (project no. 20224CRB9E, CUP B53C24006310006). We also acknowledge funding from the Dutch Research Council (NWO) through a Vidi grant (grant ID: https://doi.org/10.61686/SSXQW87450).

## Conflicts of Interest

The authors declare no conflicts of interest.

## Supporting information


**Data S1:** sim70774‐sup‐0001‐Supinfo.pdf.

## Data Availability

The data that support the findings of this study are available at https://search.r‐project.org/CRAN/refmans/joineRML/html/renal.html.

## References

[1] T. M. Therneau and P. M. Grambsch , Modeling Survival Data: Extending the Cox Model (Springer New York, 2000), 350.

[2] M. S. Wulfsohn and A. A. Tsiatis , “A Joint Model for Survival and Longitudinal Data Measured With Error,” Biometrics 53, no. 1 (1997): 330–339. 9147598

[3] D. Rizopoulos , “Joint Modeling of Longitudinal and Time‐To‐Event Data: Challenges and Future Directions,” in Advances in Theoretical and Applied Statistics. Studies in Theoretical and Applied Statistics(), ed. N. Torelli , F. Pesarin , and A. Bar‐Hen (Springer, 2013).

[4] H. C. van Houwelingen , “Dynamic Prediction by Landmarking in Event History Analysis,” Scandinavian Journal of Statistics 34, no. 1 (2007): 70–85.

[5] K. Li and S. Luo , “Dynamic Prediction of Alzheimer's Disease Progression Using Features of Multiple Longitudinal Outcomes and Time‐To‐Event Data,” Statistics in Medicine 38, no. 24 (2019): 4804–4818. 10.1002/sim.8334 PMC6800781 31386218

[6] D. Gomon , H. Putter , M. Fiocco , and M. Signorelli , “Dynamic Prediction of Survival Using Multivariate Functional Principal Component Analysis: A Strict Landmarking Approach,” Statistical Methods in Medical Research 33, no. 2 (2024): 256–272. 10.1177/09622802231224631 PMC10928955 38196243

[7] J. Lin , K. Li , and S. Luo , “Functional Survival Forests for Multivariate Longitudinal Outcomes: Dynamic Prediction of Alzheimer's Disease Progression,” Statistical Methods in Medical Research 30, no. 1 (2021): 99–111. 10.1177/0962280220941532 PMC7855476 32726189

[8] A. Devaux , C. Proust‐Lima , and R. Geneur , “Random Forests for Time‐Fixed and Time‐Dependent Predictors: The DynForest R Package,” (2023) arXiv:230202670 [statML].

[9] M. Signorelli , P. Spitali , C. Al‐Khalili Szigyarto , MARK‐MD Consortium , and R. Tsonaka , “Penalized Regression Calibration: A Method for the Prediction of Survival Outcomes Using Complex Longitudinal and High‐Dimensional Data,” Statistics in Medicine 40, no. 27 (2021): 6178–6196. 10.1002/sim.9178 PMC9293191 34464990

[10] M. Signorelli , “Pencal: An R Package for the Dynamic Prediction of Survival With Many Longitudinal Predictors,” R Journal 16, no. 2 (2025): 134–153.

[11] M. Amico and I. van Keilegom , “Cure Models in Survival Analysis,” Annual Review of Statistics and Its Application 5 (2018): 311–342.

[12] J. W. Boag , “Maximum Likelihood Estimates of the Proportion of Patients Cured by Cancer Therapy,” Journal of the Royal Statistical Society. Series B, Statistical Methodology 11, no. 1 (1949): 15–44.

[13] J. Berkson and R. P. Gage , “Survival Curve for Cancer Patients Following Treatment,” Journal of the American Statistical Association 47, no. 259 (1952): 501–515.

[14] A. Y. Yakovlev and A. D. Tsodikov , “Stochastic Models of Tumor Latency and Their Biostatistical Applications,” in Series in Mathematical Biology and Medicine (World Scientific, 1996), 269.

[15] H. Shi and G. Yin , “Landmark Cure Rate Models With Time‐Dependent Covariates,” Statistical Methods in Medical Research 26, no. 5 (2017): 2042–2054. 10.1177/0962280217708681 28627311

[16] P. Lambert and V. Bremhorst , “Inclusion of Time‐Varying Covariates in Cure Survival Models With an Application in Fertility Studies,” Journal of the Royal Statistical Society: Series A (Statistics in Society) 183, no. 1 (2020): 333–354.

[17] A. Beretta and C. Heuchenne , “Variable Selection in Proportional Hazards Cure Model With Time‐Varying Covariates, Application to US Bank Failures,” Journal of Applied Statistics 46, no. 9 (2018): 1529–1549.

[18] N. J. Law , J. M. Taylor , and H. Sandler , “The Joint Modeling of a Longitudinal Disease Progression Marker and the Failure Time Process in the Presence of Cure,” Biostatistics 3, no. 4 (2002): 547–563. 10.1093/biostatistics/3.4.547 12933597

[19] S. Kim , D. Zeng , Y. Li , and D. Spiegelman , “Joint Modeling of Longitudinal and Cure‐Survival Data,” Journal of Statistical Theory and Practice 7 (2013): 324–344. 10.1080/15598608.2013.772036 PMC3733282 23926445

[20] A. Barbieri and C. Legrand , “Joint Longitudinal and Time‐To‐Event Cure Models for the Assessment of Being Cured,” Statistical Methods in Medical Research 29, no. 4 (2020): 1256–1270. 10.1177/0962280219853599 31213153

[21] C. Xie , X. Huang , R. Li , A. Tsodikov , and K. Bhalla , “Individual Dynamic Prediction for Cure and Survival Based on Longitudinal Biomarkers,” Annals of Applied Statistics 18, no. 4 (2024): 2796–2817. 10.1214/24-aoas1906 PMC11864788 40017565

[22] Y. Peng and K. B. Dear , “A Nonparametric Mixture Model for Cure Rate Estimation,” Biometrics 56, no. 1 (2000): 237–243. 10.1111/j.0006-341x.2000.00237.x 10783801

[23] J. P. Sy and J. M. Taylor , “Estimation in a Cox Proportional Hazards Cure Model,” Biometrics 56, no. 1 (2000): 227–236. 10.1111/j.0006-341x.2000.00227.x 10783800

[24] C. E. McCulloch and S. R. Searle , “Generalized, Linear, and Mixed Models,” in Wiley Series in Probability and Statistics (John Wiley & Sons Inc., 2001).

[25] D. M. Berridge and R. Crouchley , Multivariate Generalized Linear Mixed Models Using R (CRC Press, 2011), 304.

[26] F. Achana , D. Gallacher , R. Oppong , et al., “Multivariate Generalized Linear Mixed‐Effects Models for the Analysis of Clinical Trial–Based Cost‐Effectiveness Data,” Medical Decision Making 41 (2021): 667–684. 10.1177/0272989X211003880 PMC8295965 33813933

[27] N. Breslow and D. Clayton , “Approximate Inference in Generalized Linear Mixed Models,” Journal of the American Statistical Association 88, no. 621 (1993): 9–25.

[28] K. A. Bollen and P. J. Curran , “Autoregressive Latent Trajectory (ALT) Models a Synthesis of Two Traditions,” Sociological Methods & Research 32, no. 3 (2004): 336–383.

[29] Y. Mundlak , “On the Pooling of Time Series and Cross Section Data,” Econometrica 46, no. 1 (1978): 69–85.

[30] R. Wolfinger and M. O'Connell , “Generalized Linear Mixed Models a Pseudo‐Likelihood Approach,” Journal of Statistical Computation and Simulation 48, no. 3–4 (1993): 233–243.

[31] A. P. Dempster , N. M. Laird , and D. B. Rubin , “Maximum Likelihood From Incomplete Data via the EM Algorithm,” Journal of the Royal Statistical Society: Series B: Methodological 39, no. 1 (1977): 1–38.

[32] N. Breslow , “Covariates Analysis of Censored Survival Data,” Biometrics 30, no. 1 (1974): 89–99. 4813387

[33] T. Louis , “Finding the Observed Information Matrix When Using the EM Algorithm,” Journal of the Royal Statistical Society: Series B: Methodological 2 (1982): 116–233.

[34] J. Asano and A. Hirakawa , “Assessing the Prediction Accuracy of a Cure Model for Censored Survival Data With Long‐Term Survivors: Application to Breast Cancer Data,” Journal of Biopharmaceutical Statistics 27, no. 6 (2017): 918–932. 10.1080/10543406.2017.1293082 28324665

[35] Y. Zhang , X. Han , and Y. Shao , “The ROC of Cox Proportional Hazards Cure Models With Application in Cancer Studies,” Lifetime Data Analysis 27, no. 2 (2021): 195–215. 10.1007/s10985-021-09516-6 33507457

[36] E. Graf , C. Schmoor , W. Sauerbrei , and M. Schumacher , “Assessment and Comparison of Prognostic Classification Schemes for Survival Data,” Statistics in Medicine 18, no. 17–18 (1999): 2529–2545. 10.1002/(sici)1097-0258(19990915/30)18:17/18<2529::aid-sim274>3.0.co;2-5 10474158

[37] P. J. Heagerty , T. Lumley , and M. S. Pepe , “Time‐Dependent ROC Curves for Censored Survival Data and a Diagnostic Marker,” Biometrics 56, no. 2 (2000): 337–344. 10.1111/j.0006-341x.2000.00337.x 10877287

[38] R. Schoop , E. Graf , and M. Schumacher , “Quantifying the Predictive Performance of Prognostic Models for Censored Survival Data With Time‐Dependent Covariates,” Biometrics 64, no. 2 (2008): 603–610. 10.1111/j.1541-0420.2007.00889.x 17764480

[39] F. E. Harrell , R. M. Califf , D. B. Pryor , K. L. Lee , and R. A. Rosati , “Evaluating the Yield of Medical Tests,” Journal of the American Medical Association 247, no. 18 (1982): 2543–2546. 7069920

[40] H. Uno , T. Cai , M. J. Pencina , R. B. D'Agostino , and L. J. Wei , “On the C‐Statistics for Evaluating Overall Adequacy of Risk Prediction Procedures With Censored Survival Data,” Statistics in Medicine 30, no. 10 (2011): 1105–1117. 10.1002/sim.4154 PMC3079915 21484848

[41] O. Siddiqui and M. W. Ali , “A Comparison of the Random‐Effects Pattern Mixture Model With Last‐Observation‐Carried‐Forward(Locf) Analysis in Longitudinal Clinical Trials With Dropouts,” Journal of Biopharmaceutical Statistics 8, no. 4 (1998): 545–563. 10.1080/10543409808835259 9855033

[42] M. G. Kenward and G. Molenberghs , “Last Observation Carried Forward: A Crystal Ball?,” Journal of Biopharmaceutical Statistics 19, no. 5 (2009): 872–888. 10.1080/10543400903105406 20183449

[43] G. Hickey , P. Philipson , A. Jorgensen , et al., “joineRML: Joint Modelling of Multivariate Longitudinal Data and Time‐to‐Event Outcomes,” (2025), R Package Version 0.4.6.

[44] R. Wolfe , V. Ashby , E. Milford , et al., “Comparison of Mortality in All Patients on Dialysis, Patients on Dialysis Awaiting Transplantation, and Recipients of a First Cadaveric Transplant,” New England Journal of Medicine 341, no. 23 (1999): 1725–1730. 10.1056/NEJM199912023412303 10580071

[45] K. Lamb , S. Lodhi , and H. Meier‐Kriesche , “Long‐Term Renal Allograft Survival in the United States: A Critical Reappraisal,” American Journal of Transplantation 11, no. 3 (2011): 450–462. 10.1111/j.1600-6143.2010.03283.x 20973913

[46] D. Rizopoulos and P. Ghosh , “A Bayesian Semiparametric Multivariate Joint Model for Multiple Longitudinal Outcomes and a Time‐To‐Event,” Statistics in Medicine 30, no. 12 (2011): 1366–1380. 10.1002/sim.4205 21337596

[47] S. Fieuws , G. Verbeke , B. Maes , and Y. Vanrenterghem , “Predicting Renal Graft Failure Using Multivariate Longitudinal Profiles,” Biostatistics 9, no. 3 (2008): 419–431. 10.1093/biostatistics/kxm041 18056686

