# Supplementary material for: Dynamic Prediction in Mixture Cure Models: A Model‐Based Landmarking Approach
Source: Stat Med. 2026 Oct 18;45(23-24):e70774. doi: 10.1002/sim.70774 (PMC13678029; doi:10.1002/sim.70774)
Supplement: Supplementary file 1 [file SIM-45-0-s001.pdf]

Dynamic Prediction in Mixture Cure Models: A Model-Based Landmarking Approach
Supporting Information Materials

EM Algorithm for the Landmark Mixture Cure Model

This section provides the full estimation details for a landmark mixture cure model. The procedure follows the standard Cox proportional hazards mixture cure model methodology, with estimation performed separately at each landmark time and conditional on survival up to t_ℓ.

Complete-data likelihood

At landmark time t_ℓ, let us consider the m_ℓ individuals in the risk set $\mathscr{R}(t_\ell)$, and denote by G_i the latent indicator of susceptibility. The complete-data likelihood is

$$\mathscr{L}_c(\theta) = \prod_{i=1}^{m_\ell} [\pi(\mathbf{x}_i) f_u(T_i \mid \mathbf{z}_i, \hat{s}(\mathbf{y}_i))]^{\delta_i G_i} [\pi(\mathbf{x}_i) S_u(T_i \mid \mathbf{z}_i, \hat{s}(\mathbf{y}_i))]^{(1-\delta_i)G_i} [1-\pi(\mathbf{x}_i)]^{(1-\delta_i)(1-G_i)} \tag{1}$$

All likelihood contributions are conditional on units that survive up to t_ℓ, and T_i in the above equation refers to the time measured from t_ℓ onwards.

E-step

In iteration r, the posterior expectation of G_i is

$$q_i^{(r)} = \delta_i + (1-\delta_i) \frac{\pi^{(r-1)}(\mathbf{x}_i) S_u^{(r-1)}(T_i \mid \mathbf{z}_i, \hat{s}(\mathbf{y}_i))}{\left[1-\pi^{(r-1)}(\mathbf{x}_i)\right] + \pi^{(r-1)}(\mathbf{x}_i) S_u^{(r-1)}(T_i \mid \mathbf{z}_i, \hat{s}(\mathbf{y}_i))}. \tag{2}$$

M-step

The M-step maximises the expected complete-data log-likelihood, replacing G_i by $q_i^{(r)}$.
The objective function decomposes into

$$Q(\theta \mid \theta^{(r)}) = Q_1(\theta \mid \theta^{(r)}) + Q_2(\theta \mid \theta^{(r)}),$$

where

$$Q_1\left(\theta \mid \theta^{(r)}\right) = \sum_{i=1}^{m_\ell} \left[q_i^{(r)} \log \pi(\mathbf{x}_i) + (1 - q_i^{(r)})(1-\delta_i) \log(1-\pi(\mathbf{x}_i)) \right], \tag{3}$$

$$Q_2\left(\theta \mid \theta^{(r)}\right) = \sum_{i=1}^{m_\ell} \left[q_i^{(r)} \delta_i \log f_u(T_i \mid \mathbf{z}_i, \hat{s}(\mathbf{y}_i)) + q_i^{(r)} (1-\delta_i) \log S_u(T_i \mid \mathbf{z}_i, \hat{s}(\mathbf{y}_i)) \right]. \tag{4}$$

Maximisation with respect to the baseline cumulative hazard function yields the modified Breslow estimator

$$\hat{H}_0^{(r)}(t) = \sum_{i=1}^{m_\ell} \frac{I(T_i < t)\delta_i}{\sum_{j \in \mathscr{R}(t_\ell)} q_j^{(r)} \exp\left(\beta^{(r-1)\prime}\mathbf{z}_j + \psi^{(r-1)\prime}\hat{s}(\mathbf{y}_j)\right)}. \tag{5}$$

Variance estimation

Standard errors are obtained from the observed information matrix using the method proposed by Louis (1982), which provides asymptotic inference for both incidence and latency parameters within the EM framework.

Additional simulation results

To further explore potential differences, we also considered additional simulations, as detailed in the main text. Here, the process generating the latent longitudinal trajectory includes also fixed effects that are not explicitly considered as predictors in the cure survival model. Specifically, for subject i we generate the l-th longitudinal covariate, $l = 1, \dots, 4$, as

$$\eta_{il}(t) = b_{0,il} + b_{1,il} t + \gamma_l^{\prime} \mathbf{w}_{il}(t) \tag{6}$$

where $\mathbf{w}_{il}(t) = (1, t)$ and $\gamma_l = (\gamma_{0l}, \gamma_{1l})^\top$ represents fixed intercept and slope effects. The coefficients are generated as $\gamma_{0l} \sim \text{Uniform}(3, 7)$ and γ_{1l} from a symmetric $\text{Uniform}(1, 2)$ distribution with random sign.
The aim of this additional simulation scenario is to assess the robustness of the RE and PV approaches when sources of longitudinal variation are not considered in the survival component. This is only one way in which the two summaries can diverge: more generally, even when the longitudinal and survival submodels share the same covariates, RE includes the individual random effects as separate predictors, whereas PV includes only their combination at the landmark, $\hat{y}_{il}(t_\ell)$, so the two need not coincide. The present scenario is one concrete instance in which this difference becomes visible. In this setting, as shown in Figure 1 below, the PV approach moderately outperforms the RE approach, while both model-based strategies clearly outperform the LOCF method.

Importantly, these conclusions were consistent for both the balanced design and, more clearly, the unbalanced design, indicating that the comparative performance of the methods was consistent with our expectations.

$m = 300$

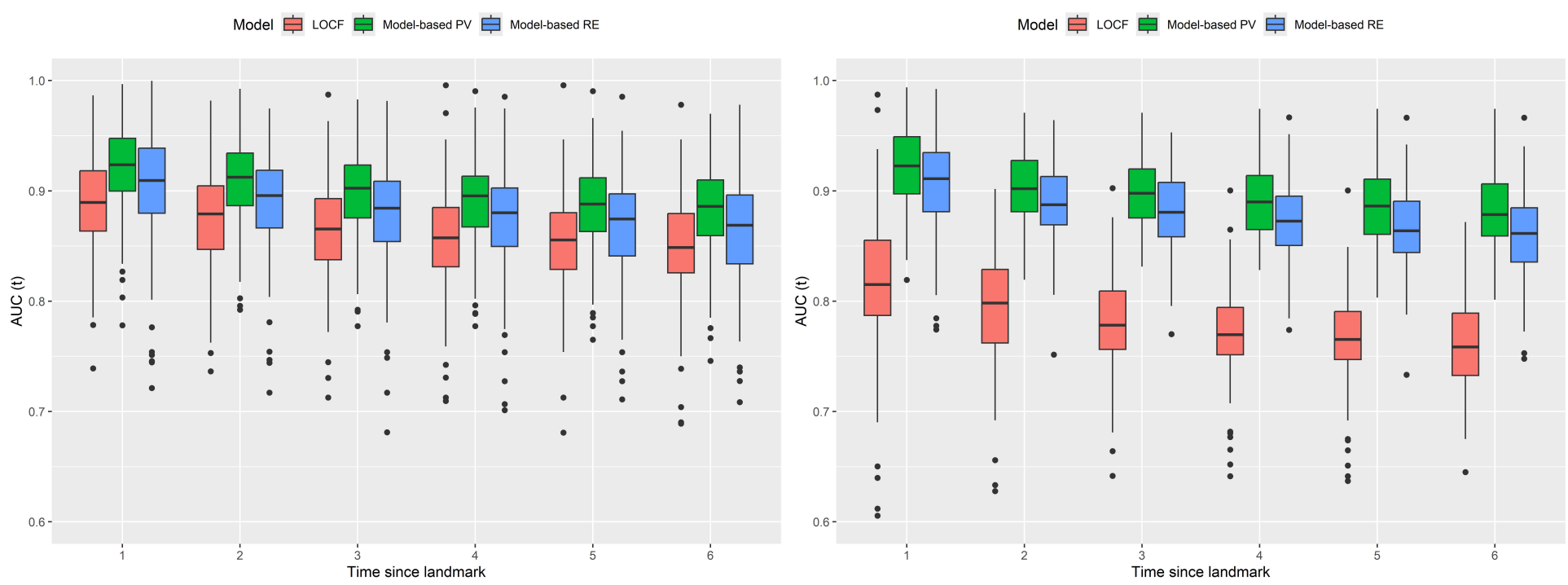

(a) Scenario 7 - 40% cured; M balanced (b) Scenario 8 - 40% cured; M unbalanced

Figure 1: Current Value Scenario, fixed effects inclusion in the data generating process.

$m = 300$

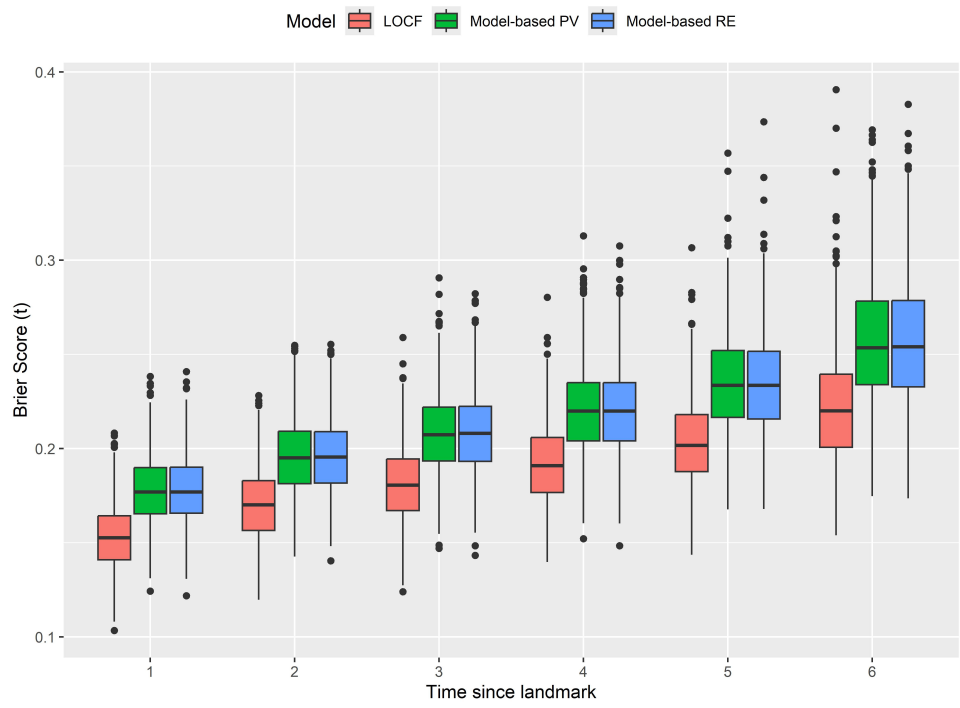

(a) Scenario 1 - 20% cured; M balanced

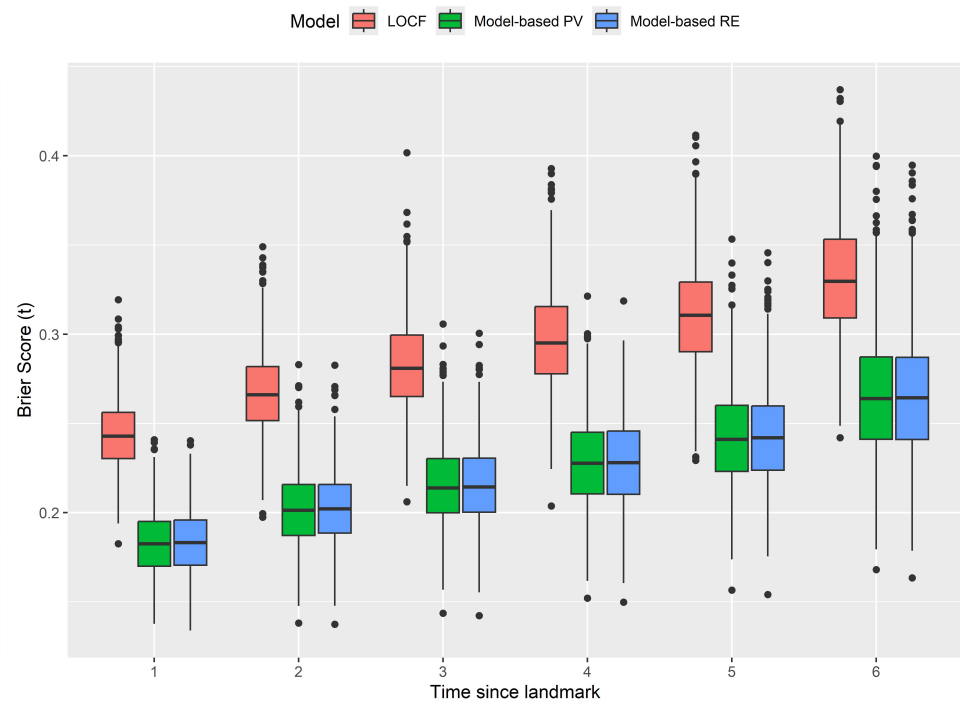

(b) Scenario 2 - 20% cured; M unbalanced

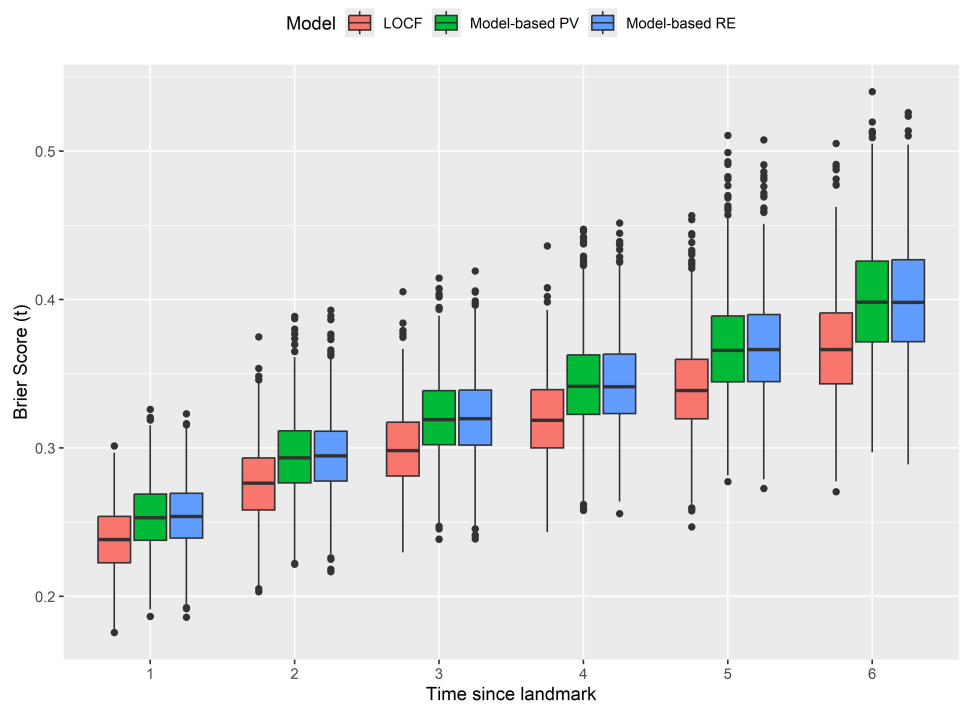

(c) Scenario 3 - 40% cured; M balanced

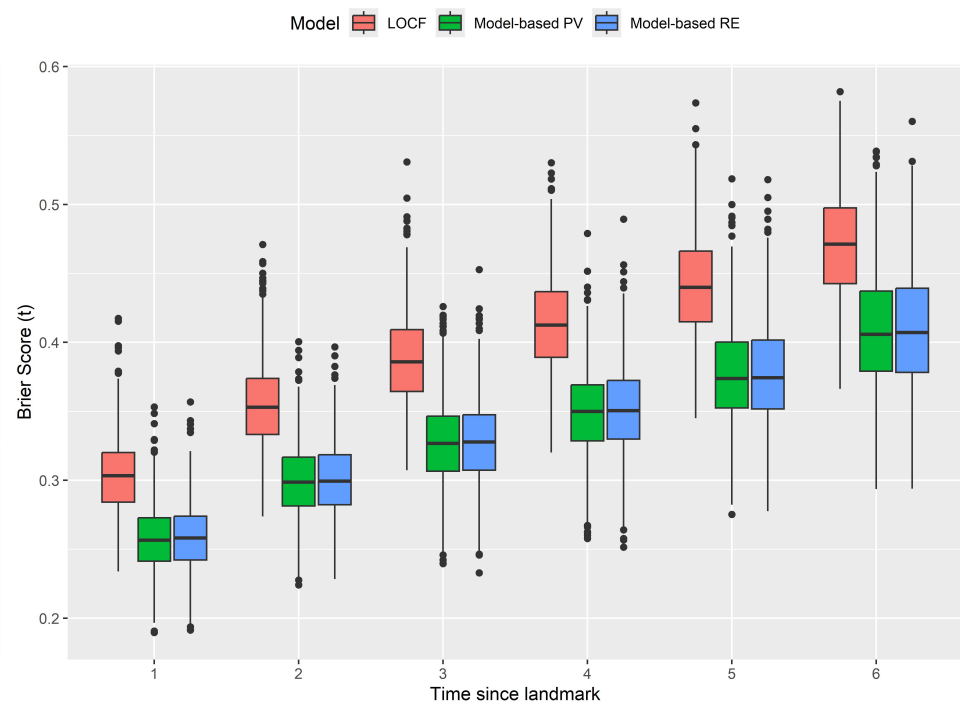

(d) Scenario 4 - 40% cured; M unbalanced

$m = 1000$

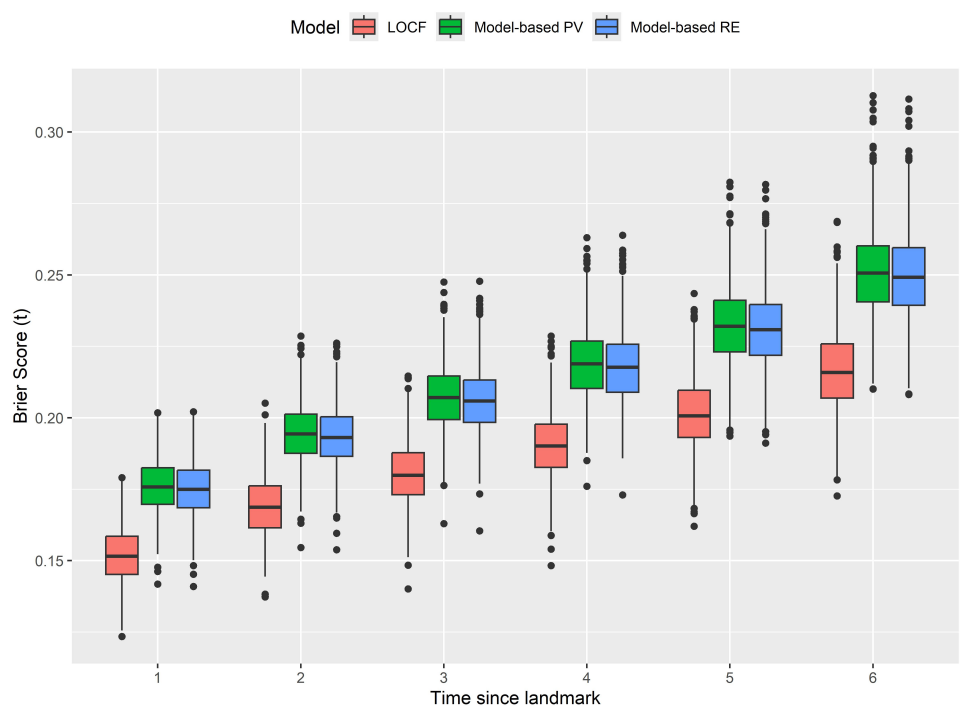

(e) Scenario 1 - 20% cured; M balanced

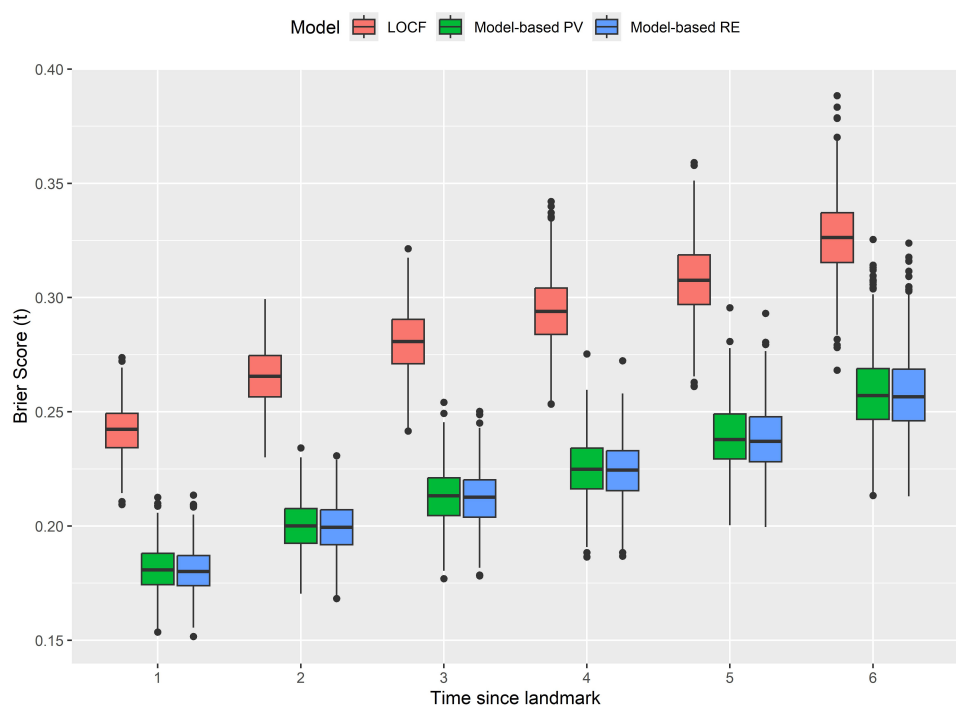

(f) Scenario 2 - 20% cured; M unbalanced

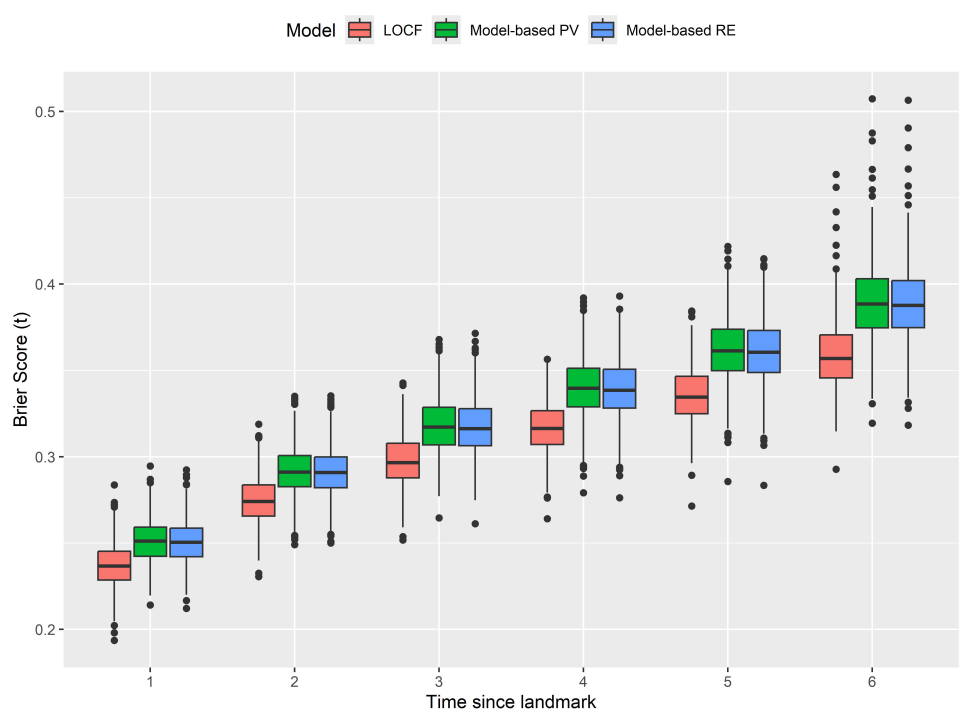

(g) Scenario 3 - 40% cured; M balanced

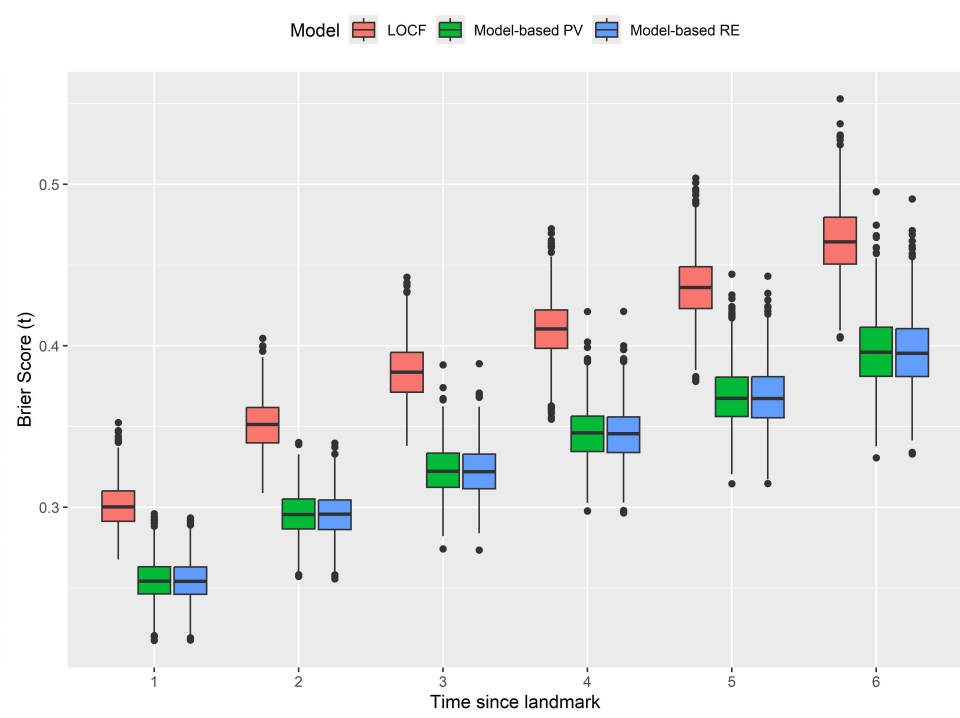

(h) Scenario 4 - 40% cured; M unbalanced

Figure 2: Time-dependent Brier Score under LOCF-based scenarios.

$m = 300$

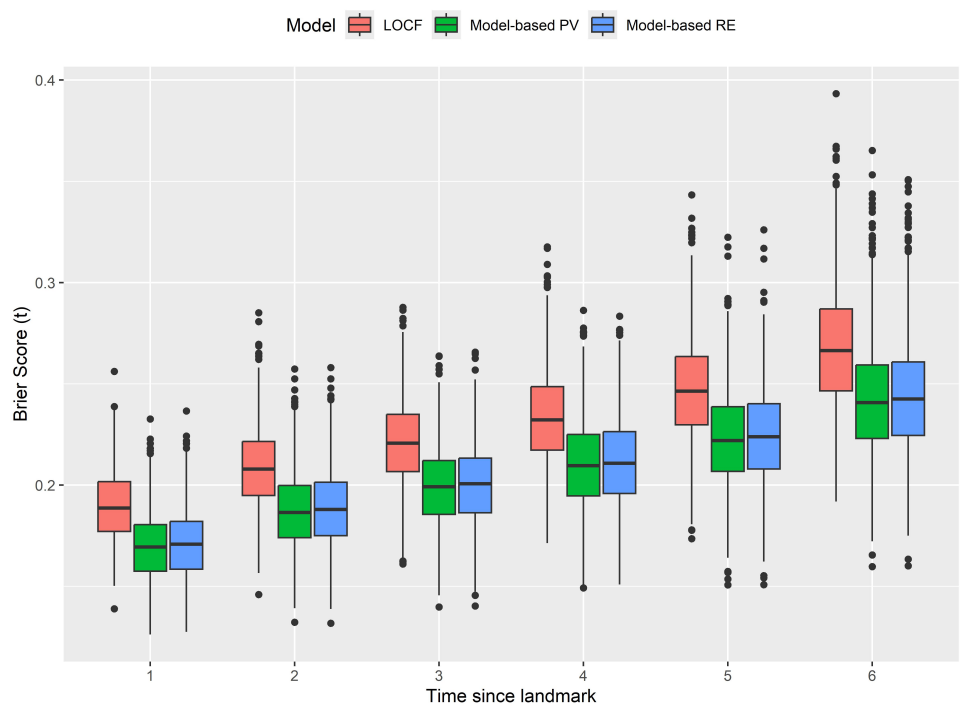

(a) Scenario 5 - 20% cured; M balanced

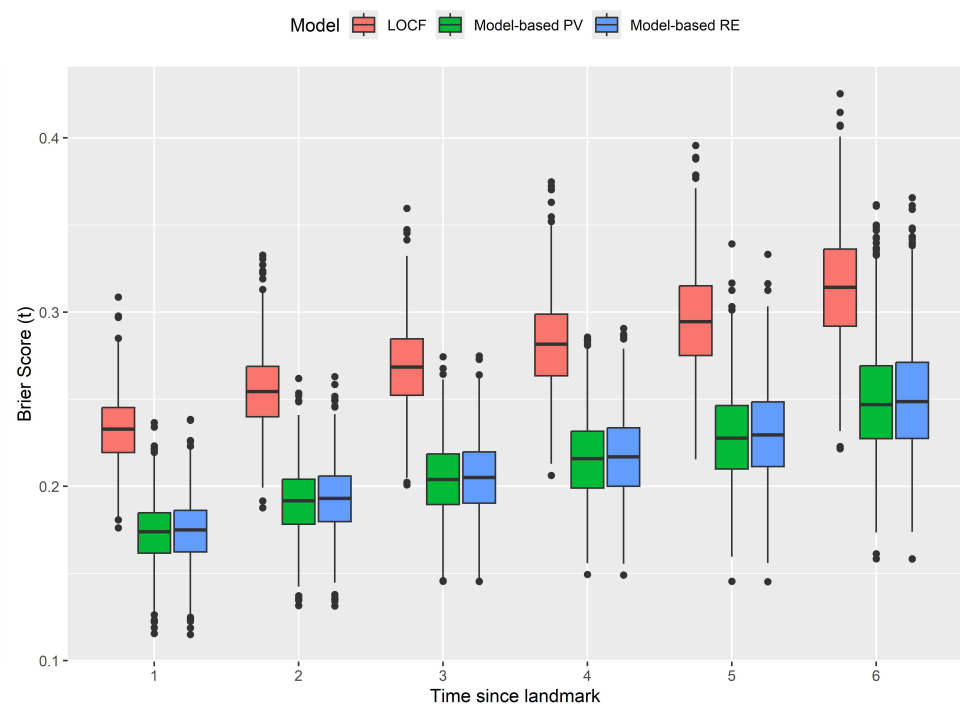

(b) Scenario 6 - 20% cured; M unbalanced

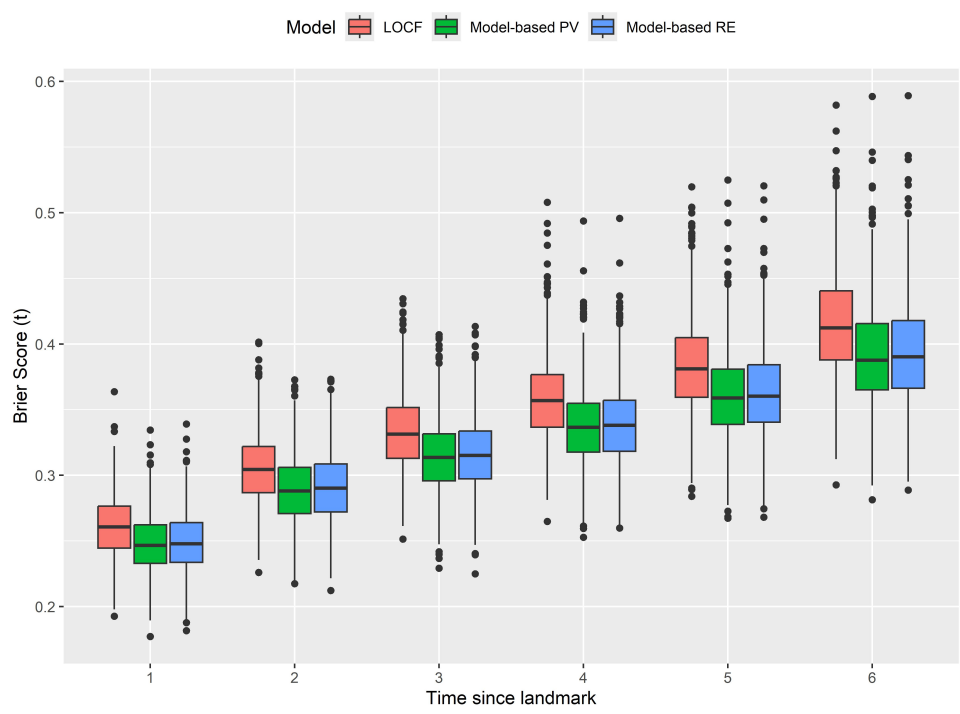

(c) Scenario 7 - 40% cured; M balanced

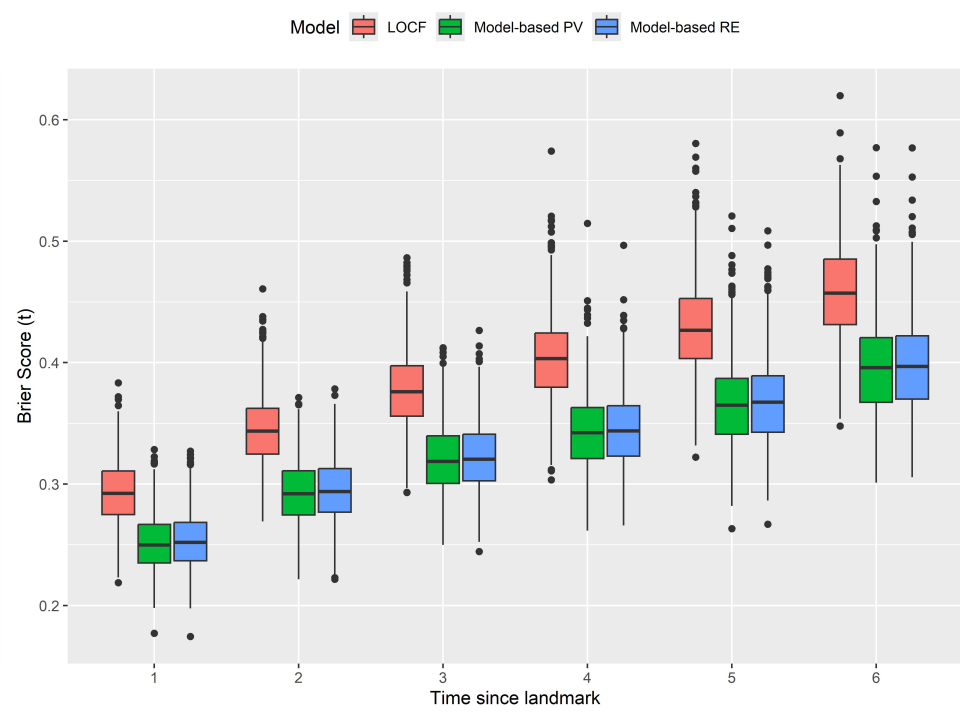

(d) Scenario 8 - 40% cured; M unbalanced

$m = 1000$

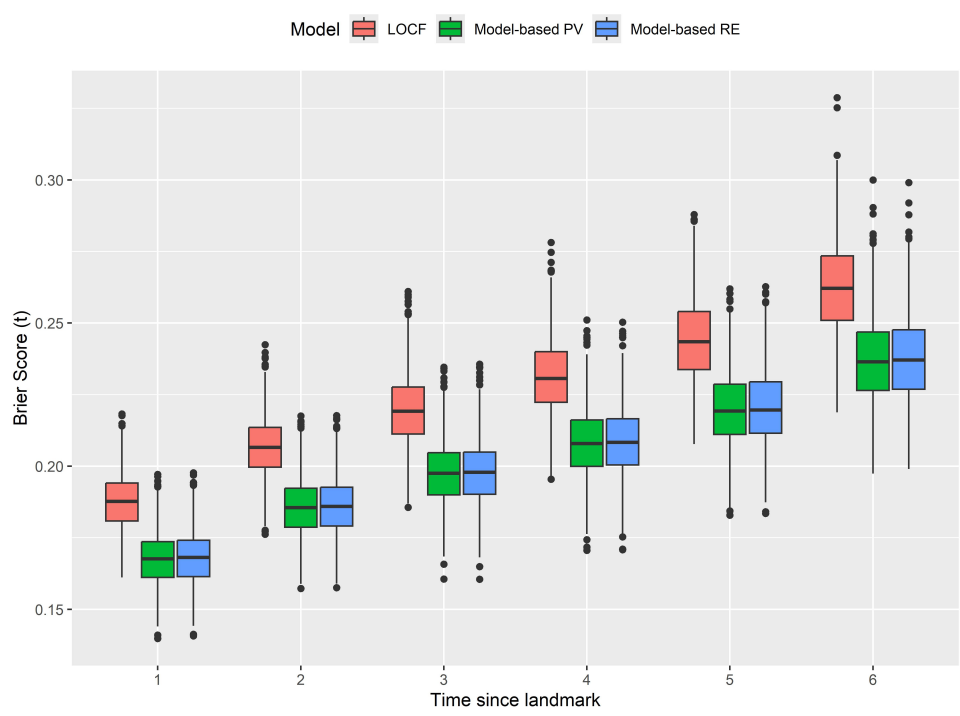

(e) Scenario 5 - 20% cured; M balanced

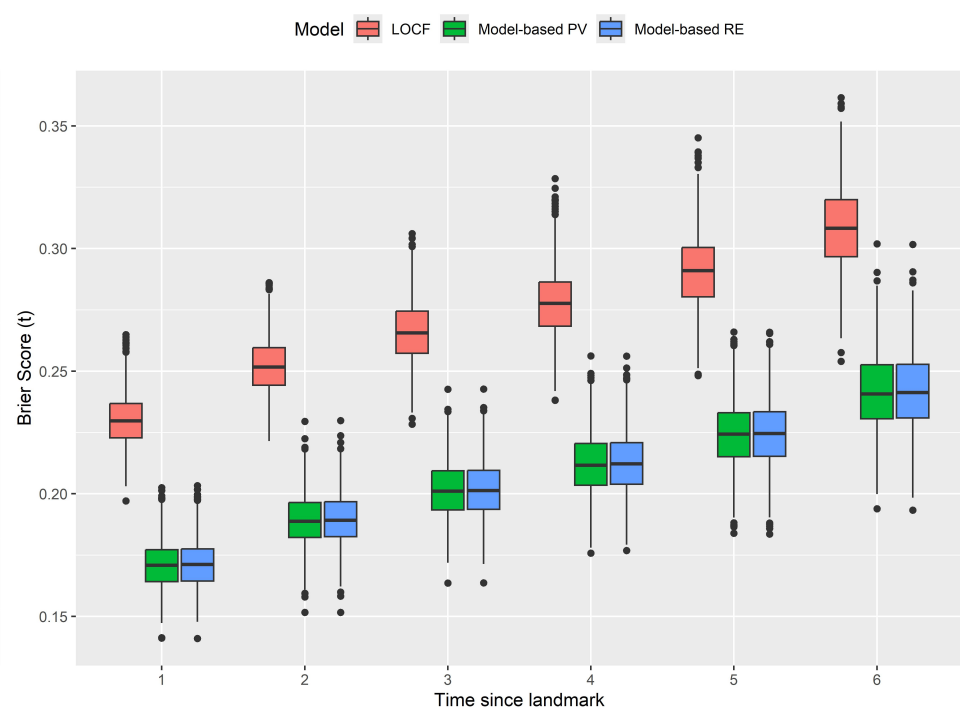

(f) Scenario 6 - 20% cured; M unbalanced

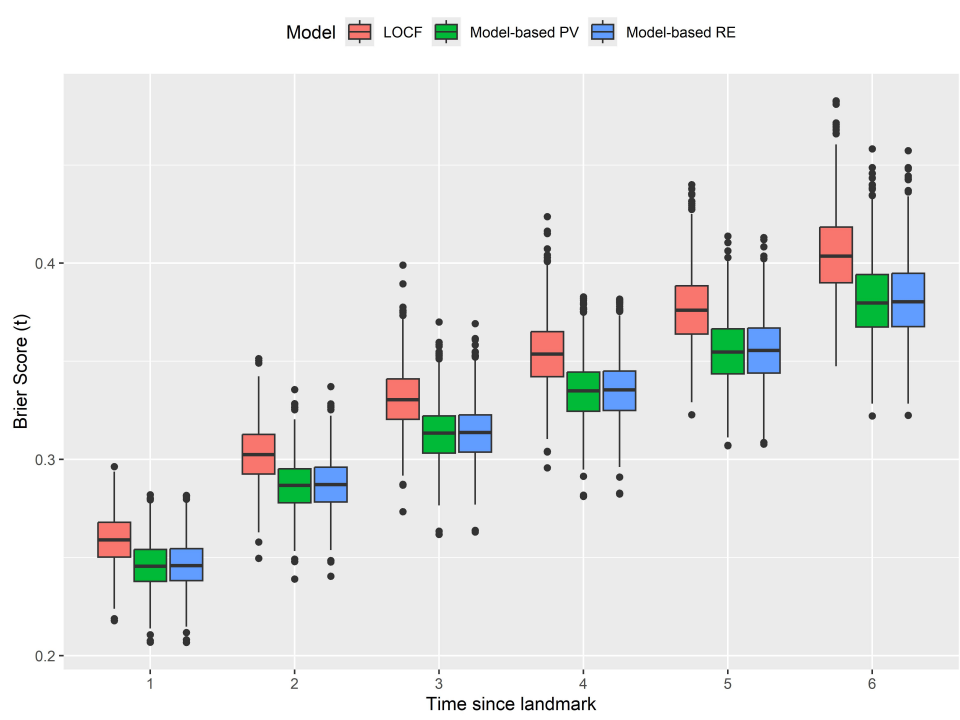

(g) Scenario 7 - 40% cured; M balanced

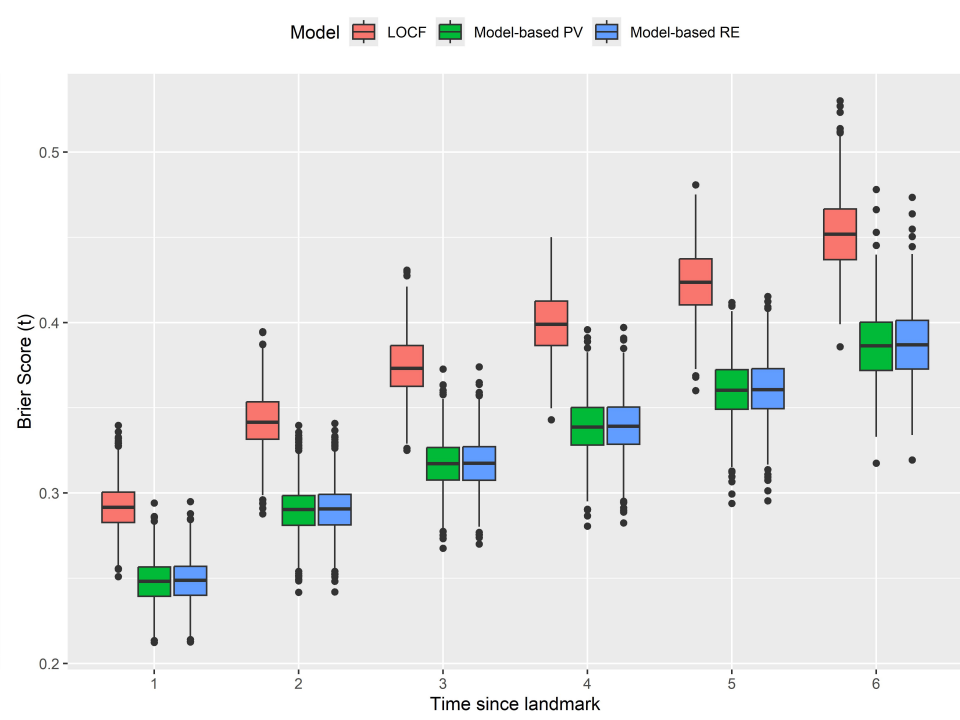

(h) Scenario 8 - 40% cured; M unbalanced

Figure 3: Time-dependent Brier Score under CV-based scenarios.

$m = 300$

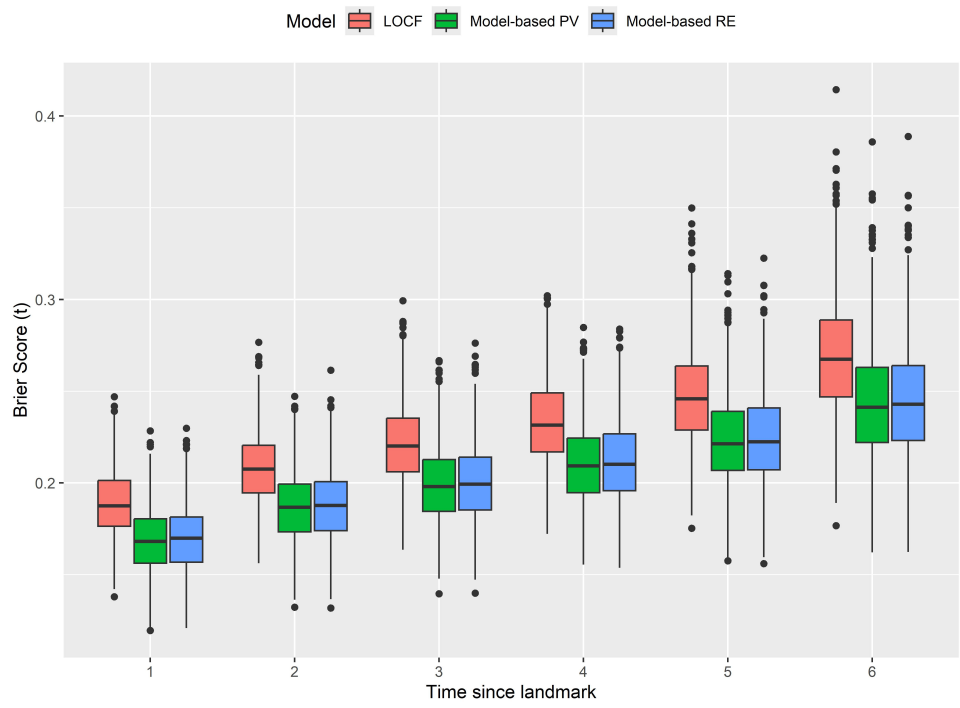

(a) Scenario 9 - 20% cured; M balanced

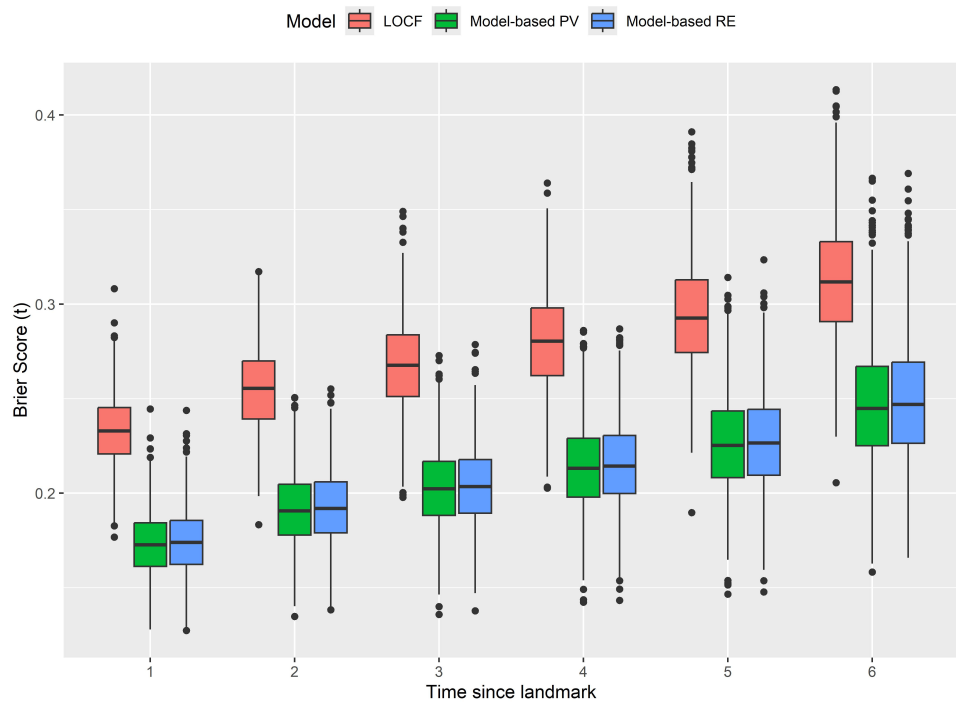

(b) Scenario 10 - 20% cured; M unbalanced

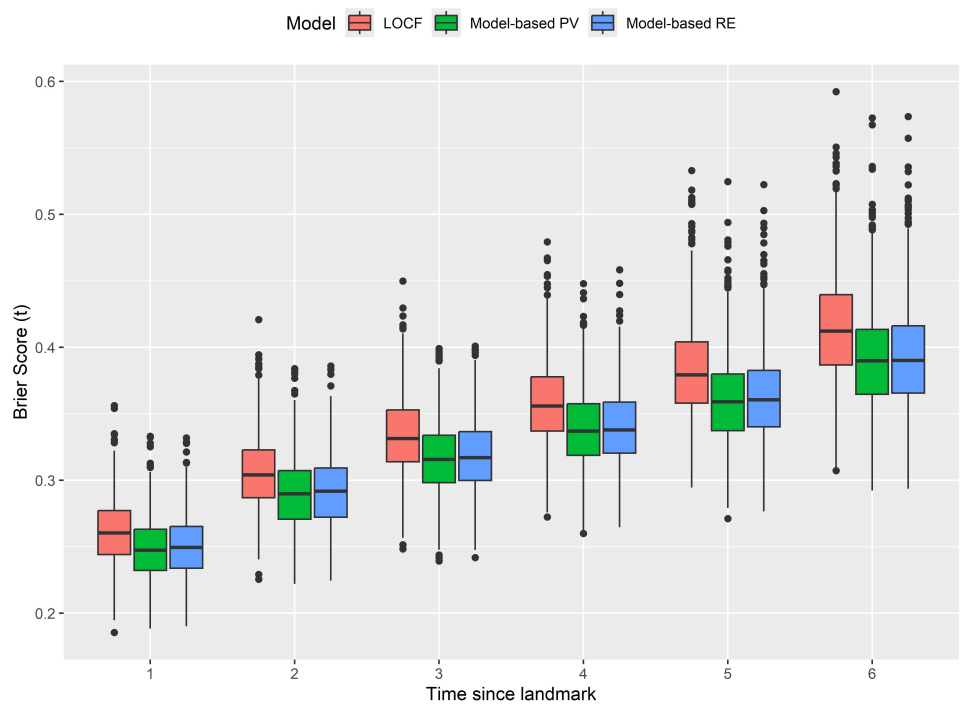

(c) Scenario 11 - 40% cured; M balanced

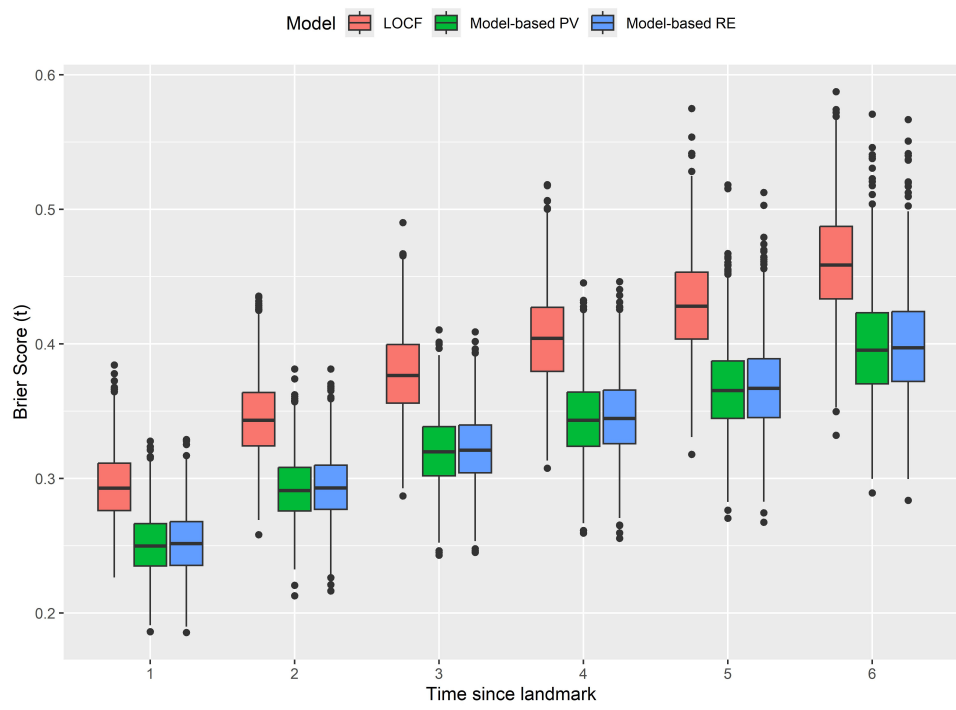

(d) Scenario 12 - 40% cured; M unbalanced

$m = 1000$

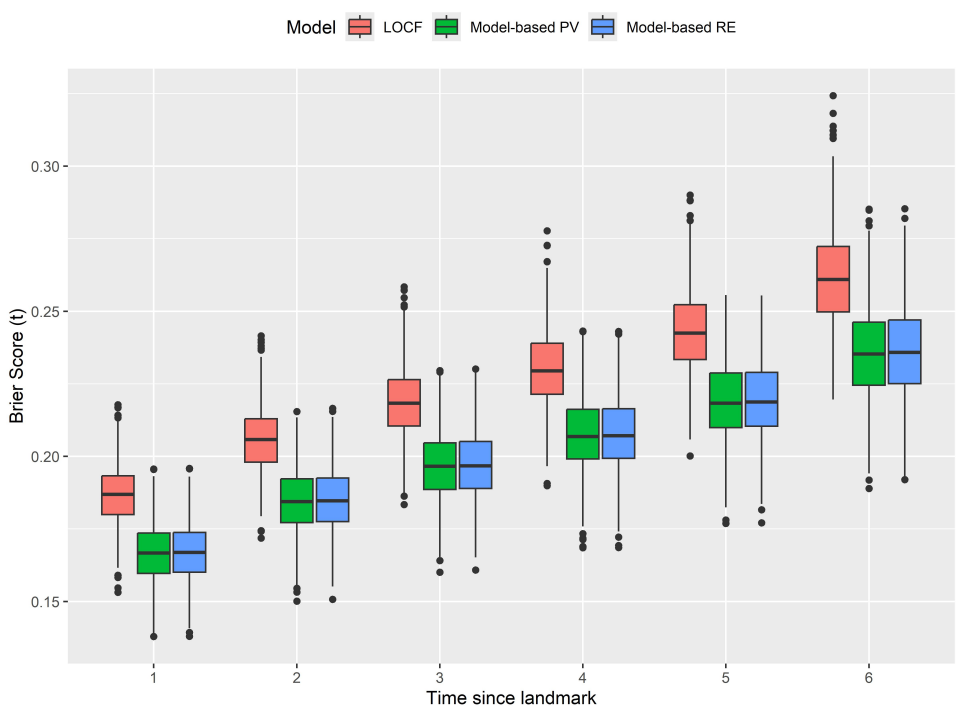

(e) Scenario 9 - 20% cured; M balanced

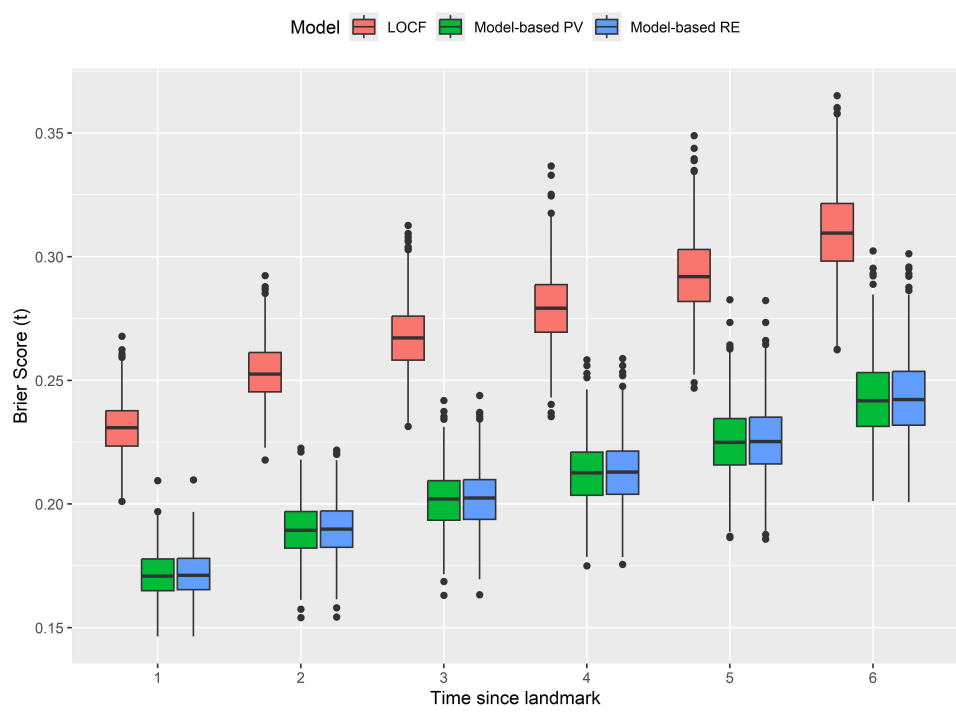

(f) Scenario 10 - 20% cured; M unbalanced

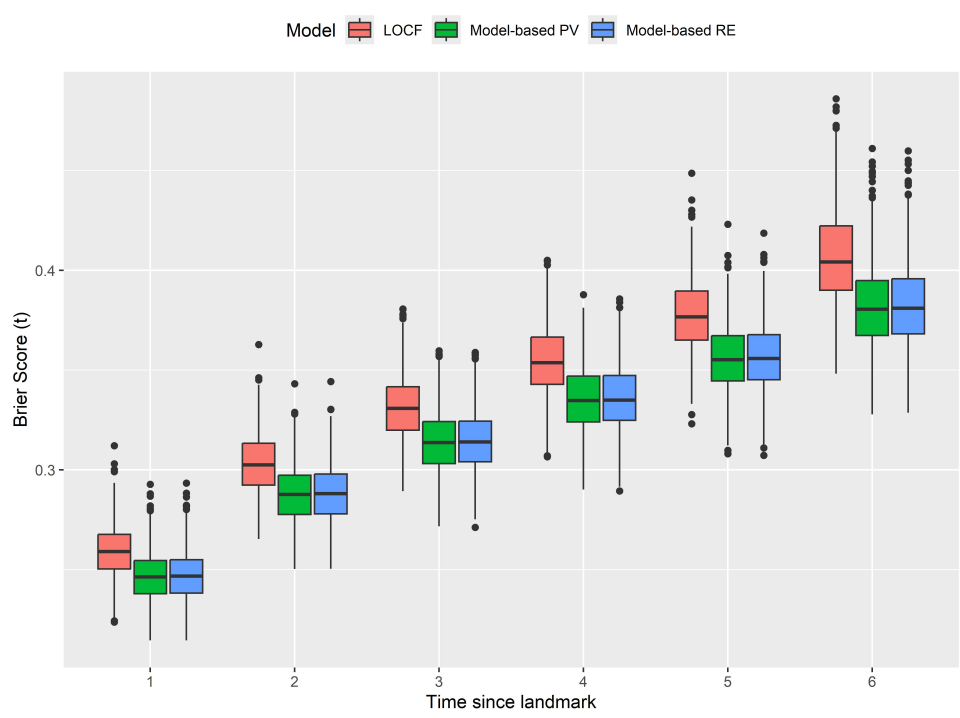

(g) Scenario 11 - 40% cured; M balanced

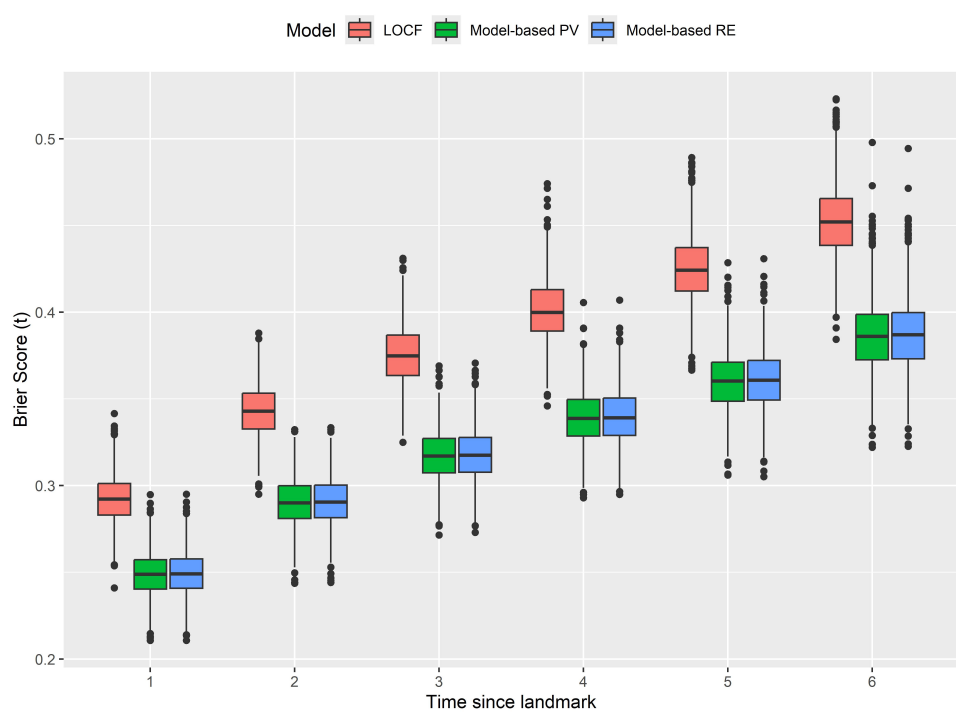

(h) Scenario 12 - 40% cured; M unbalanced

Figure 4: Time-dependent Brier Score under RE-based scenarios.

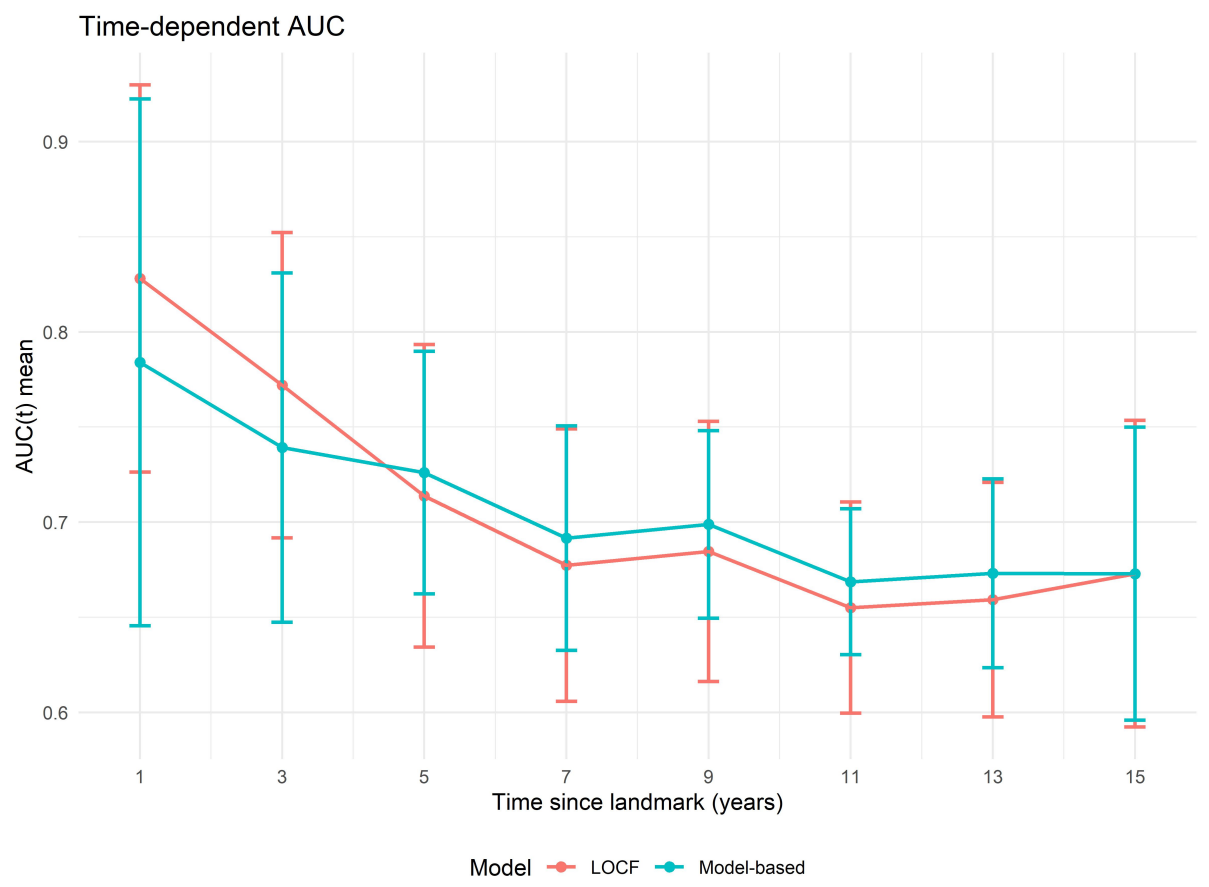

(a) Landmark time $t = 1$

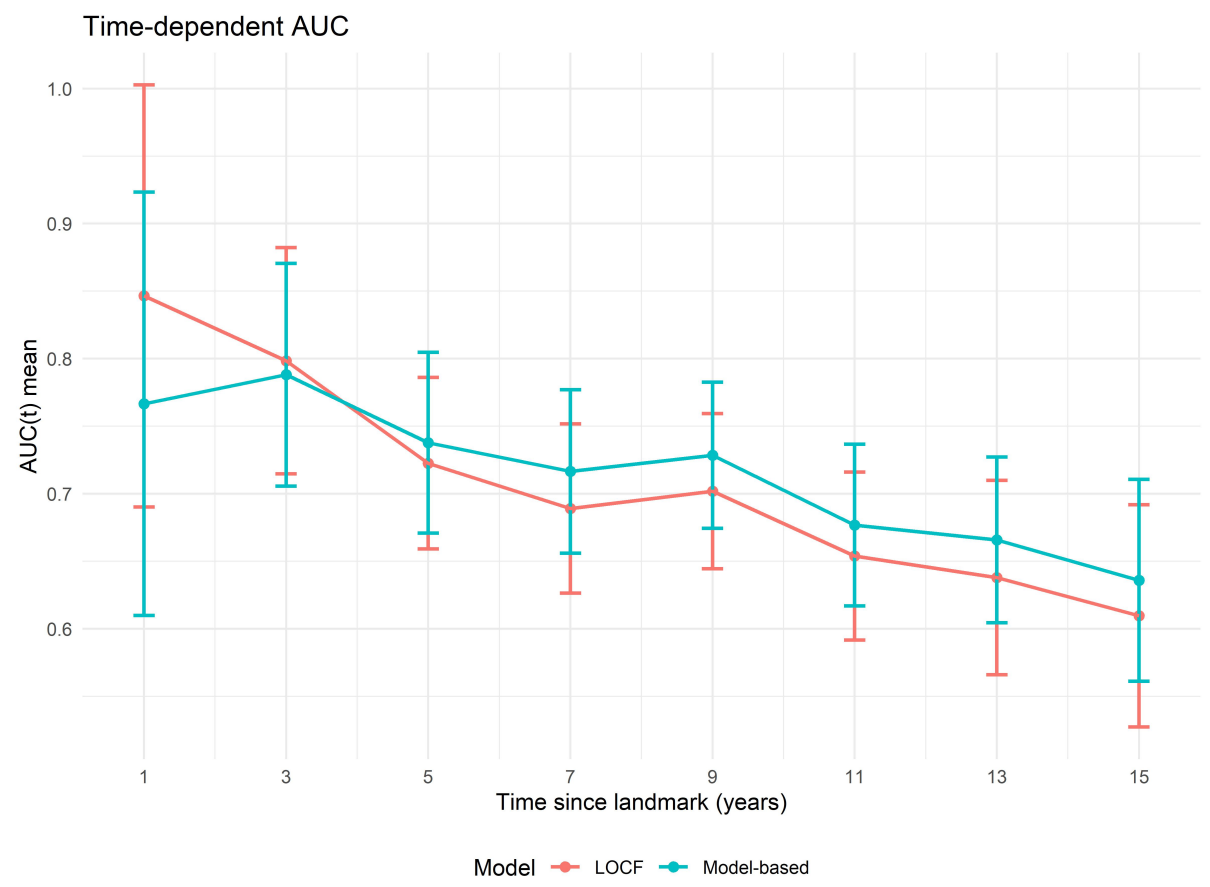

(b) Landmark time $t = 1.5$

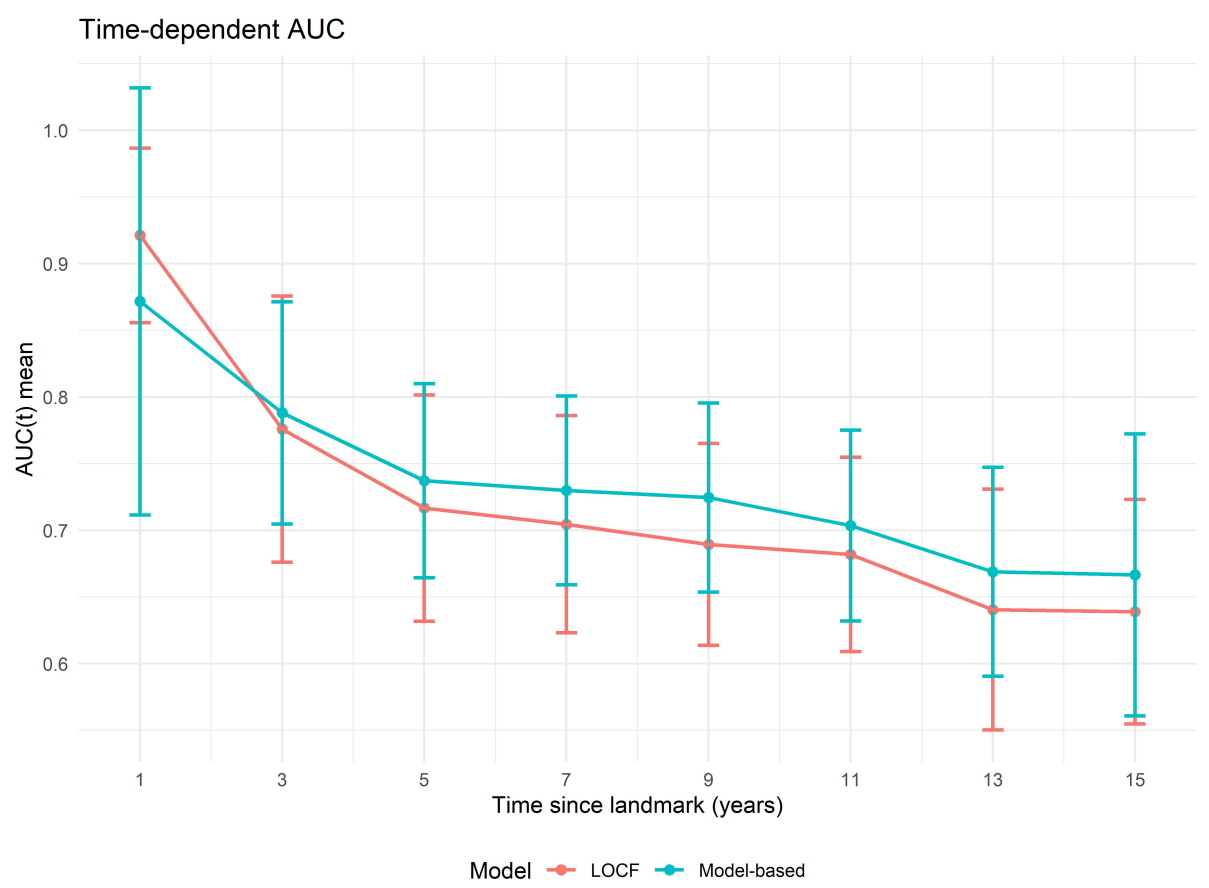

(c) Landmark time $t = 2$

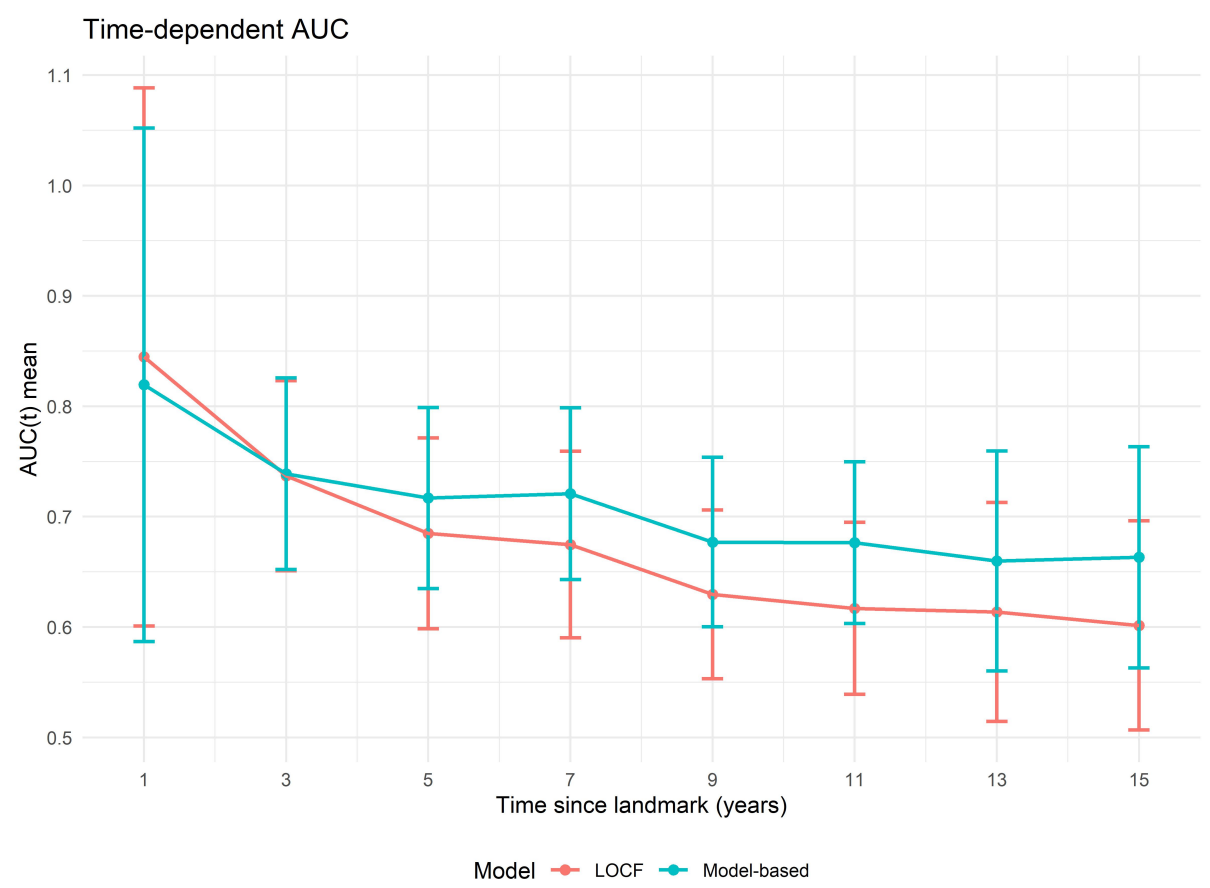

(d) Landmark time $t = 2.5$

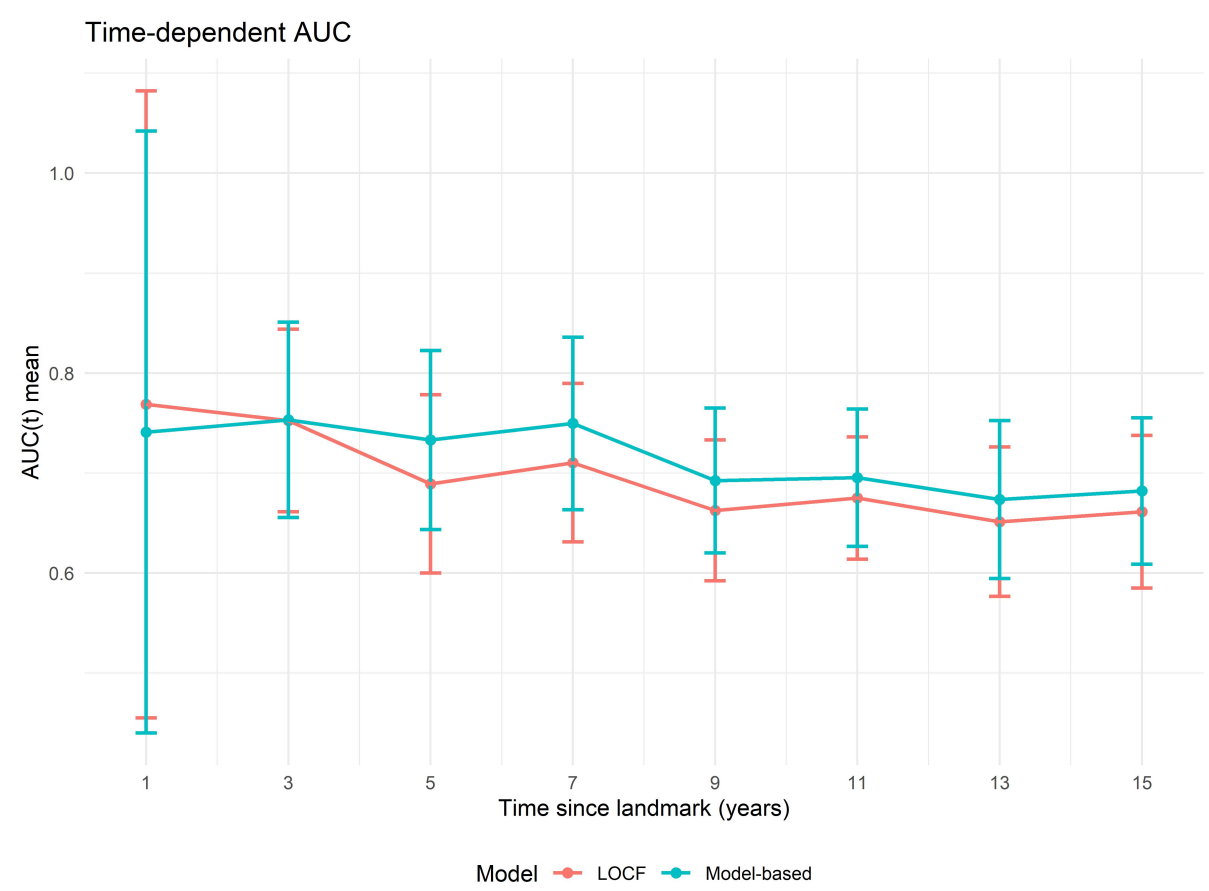

(e) Landmark time $t = 3$

Figure 5: Time-dependent AUC curves along with standard errors for the model-based and LOCF approaches at multiple landmark times.

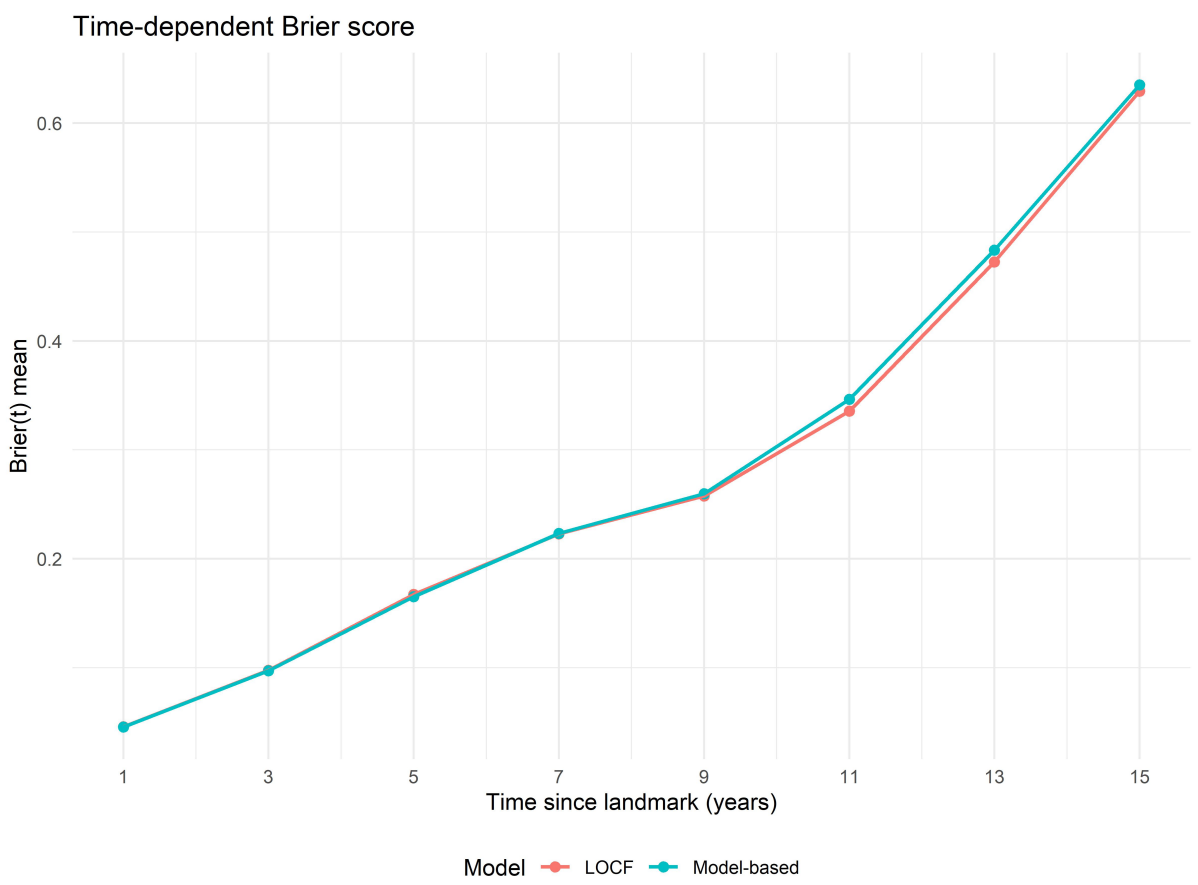

(a) Landmark time $t = 1$

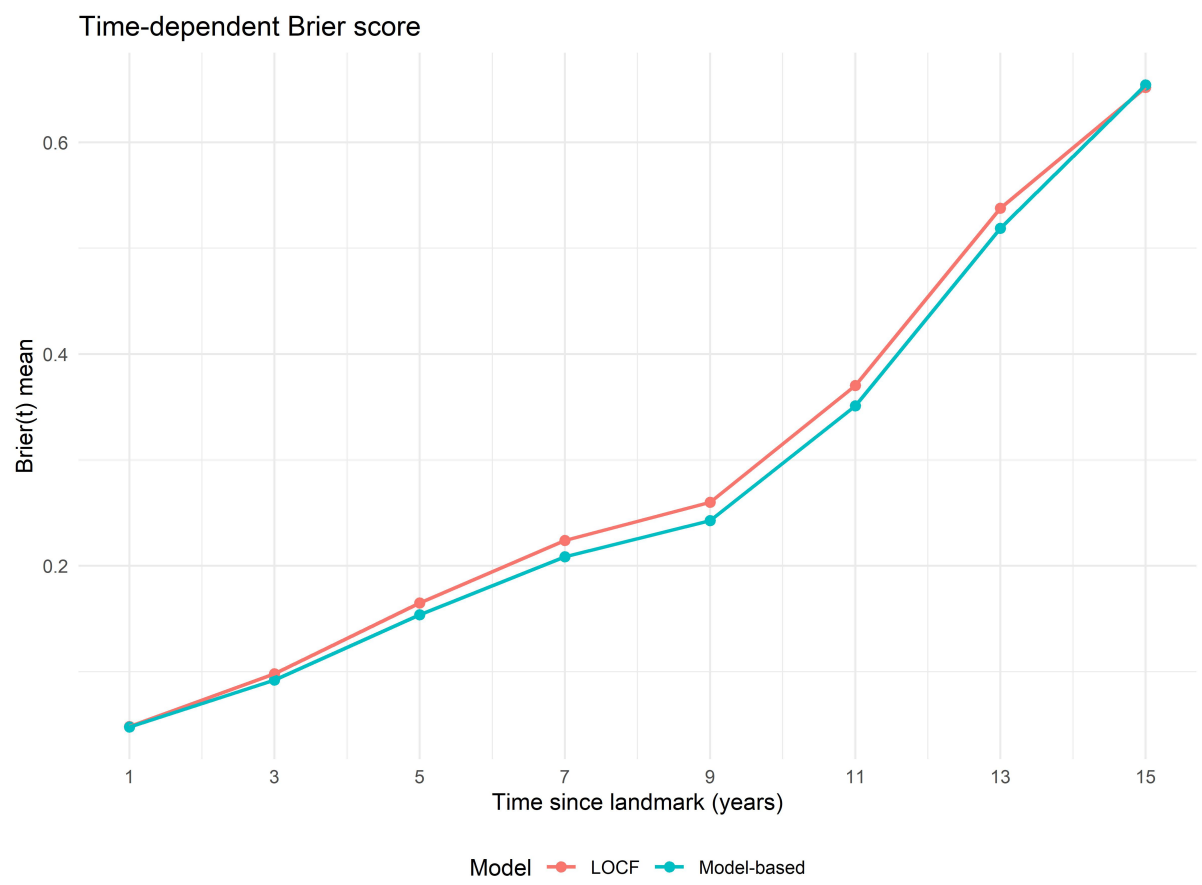

(b) Landmark time $t = 1.5$

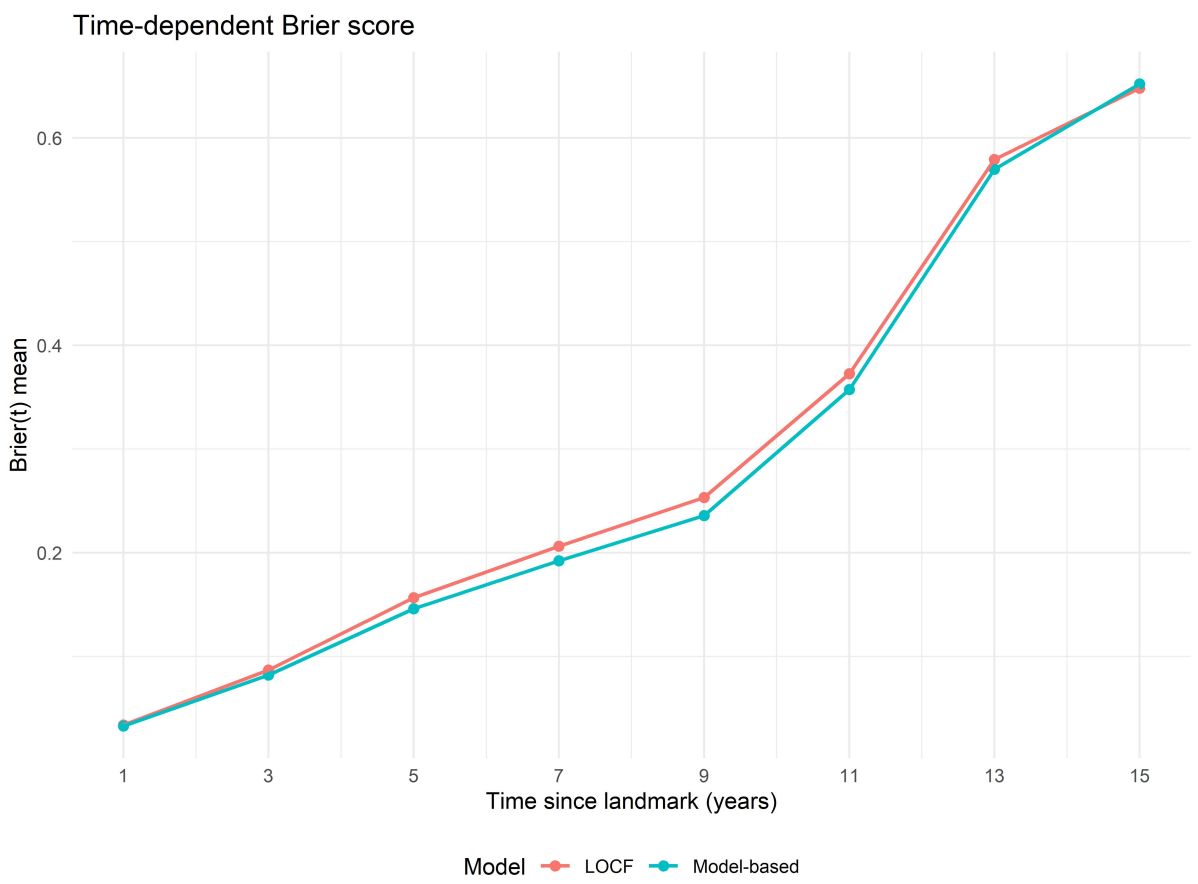

(c) Landmark time $t = 2$

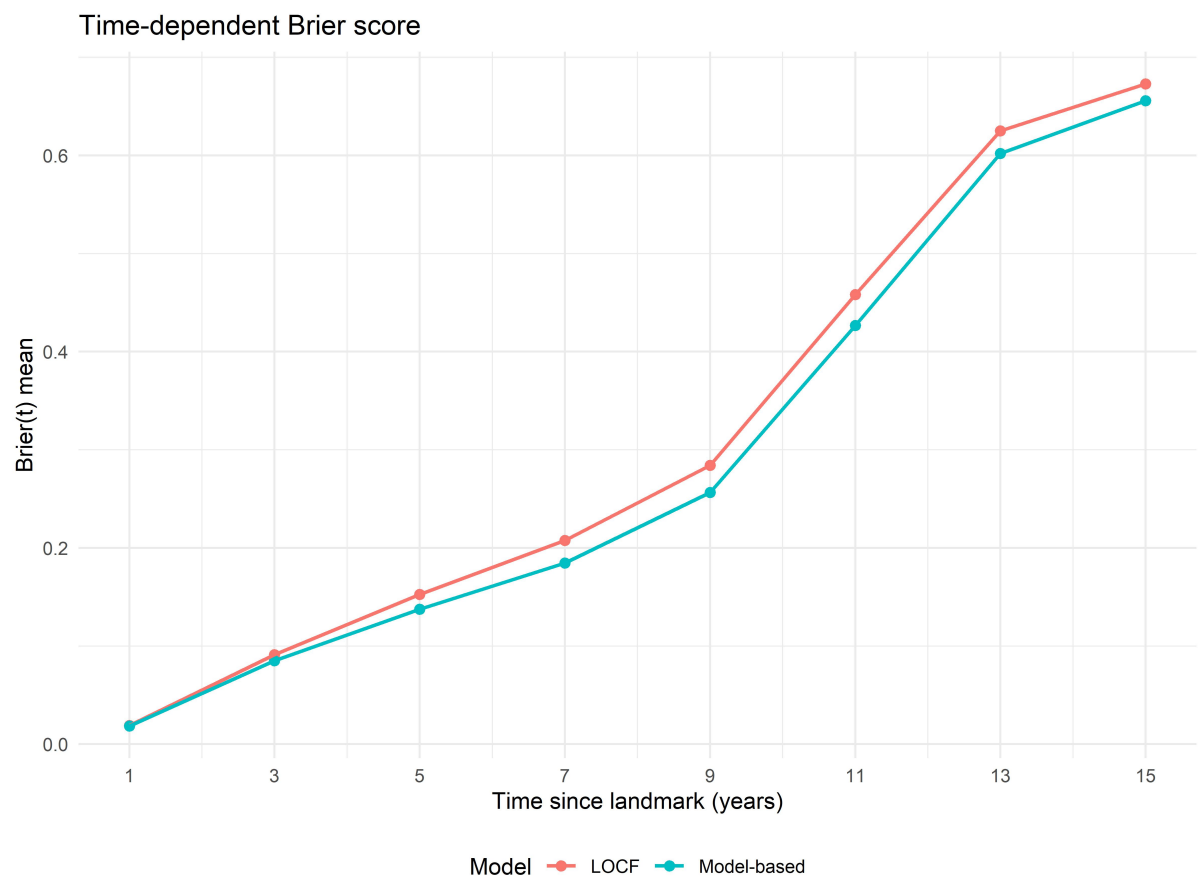

(d) Landmark time $t = 2.5$

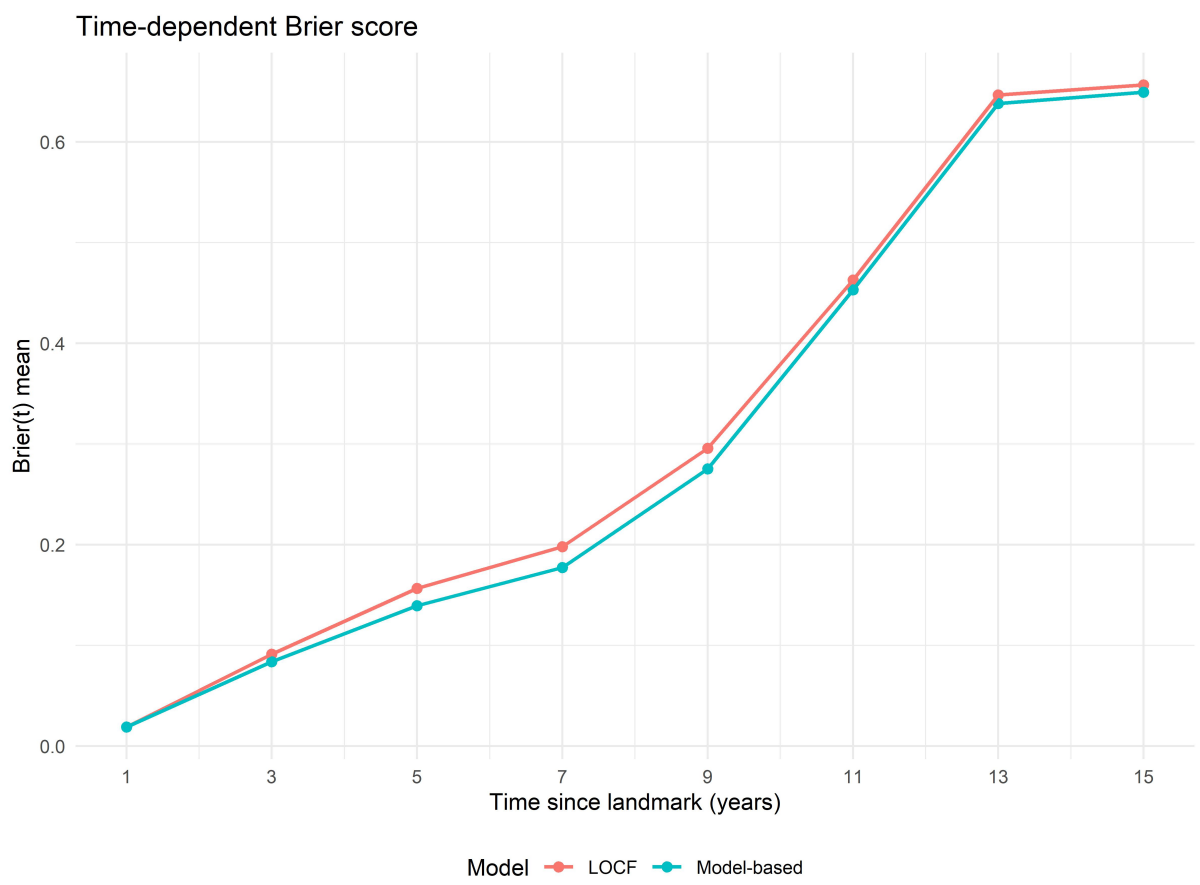

(e) Landmark time $t = 3$

Figure 6: Time-dependent Brier Score curves for the model-based and LOCF approaches at multiple landmark times.

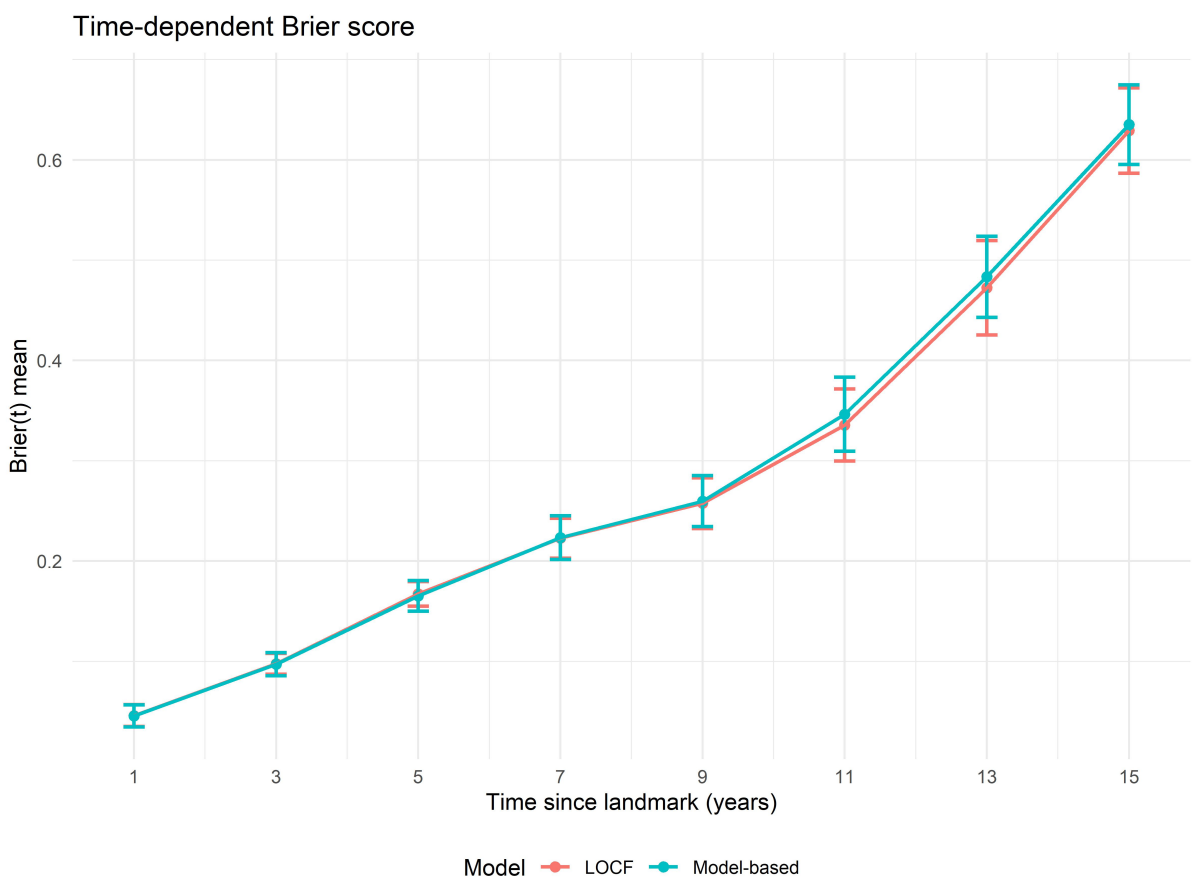

(a) Landmark time $t = 1$

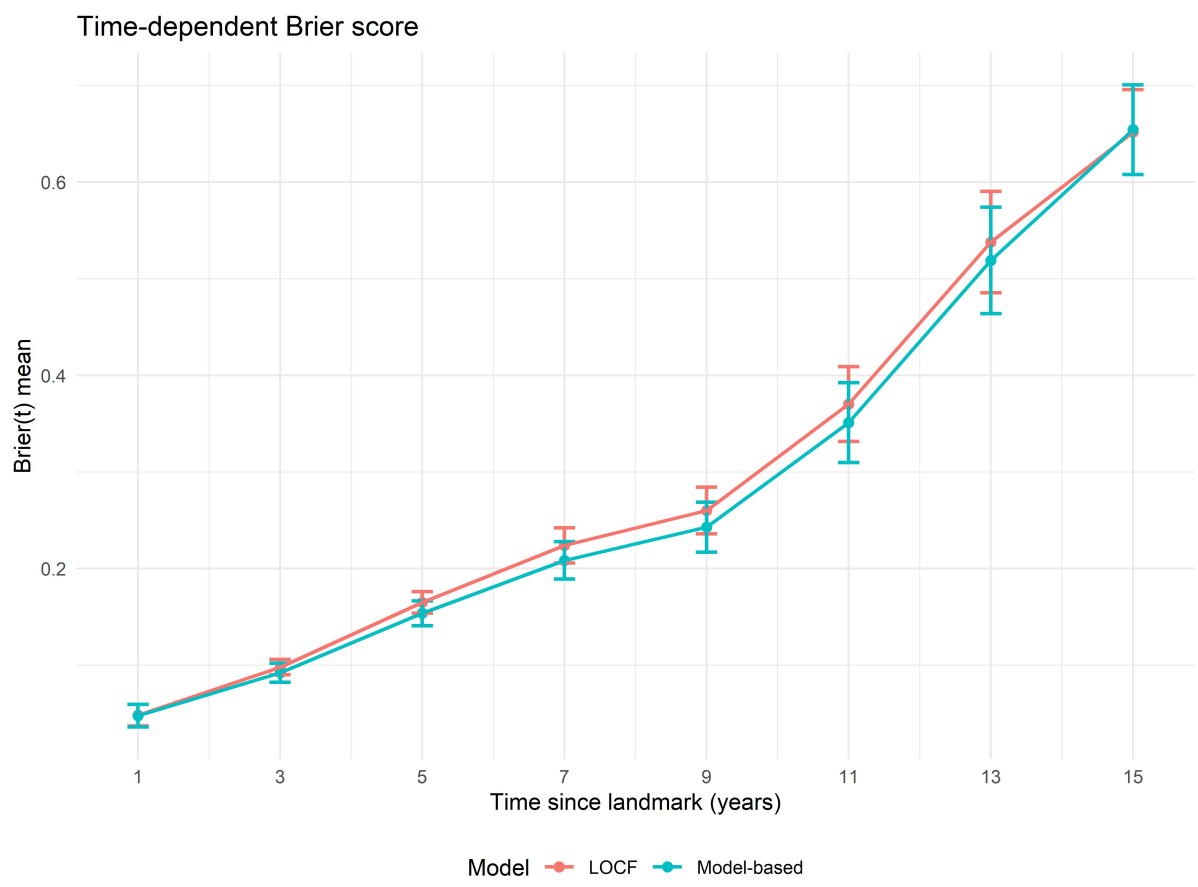

(b) Landmark time $t = 1.5$

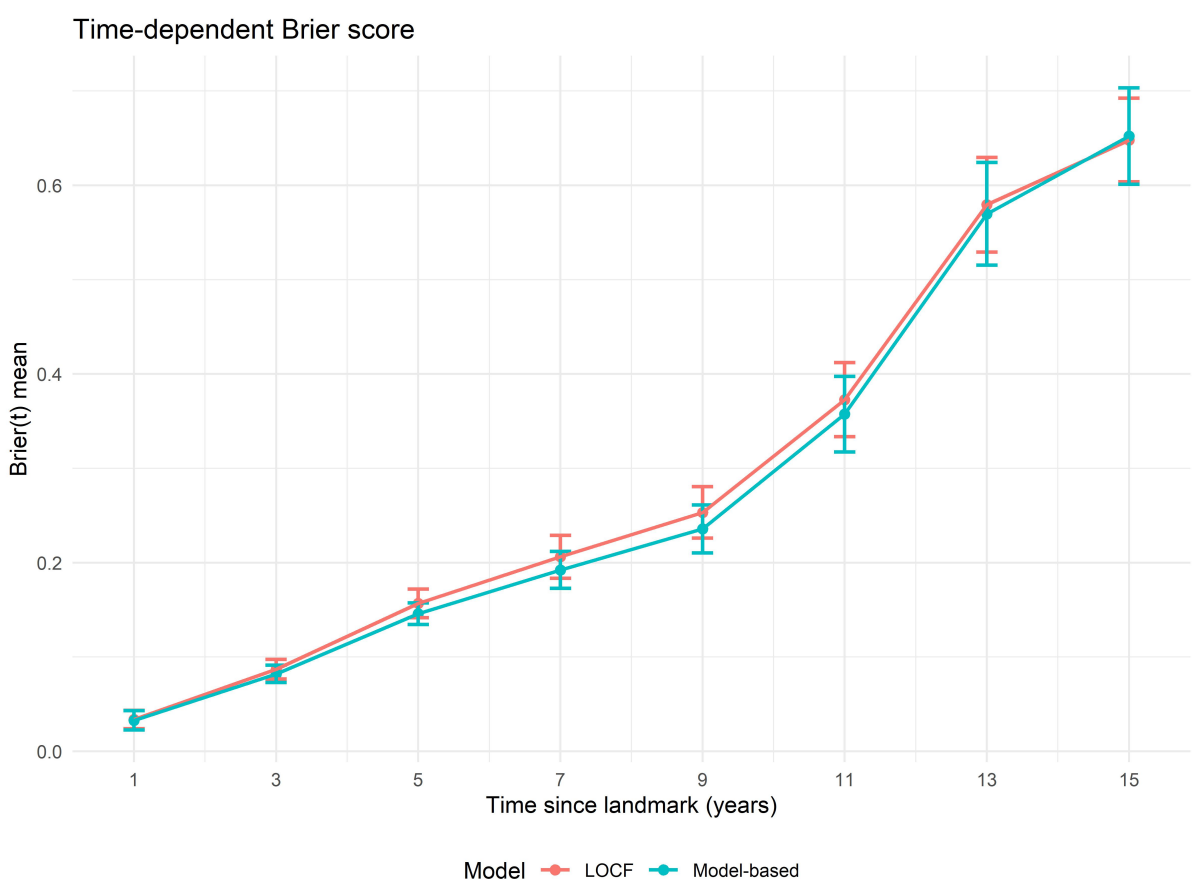

(c) Landmark time $t = 2$

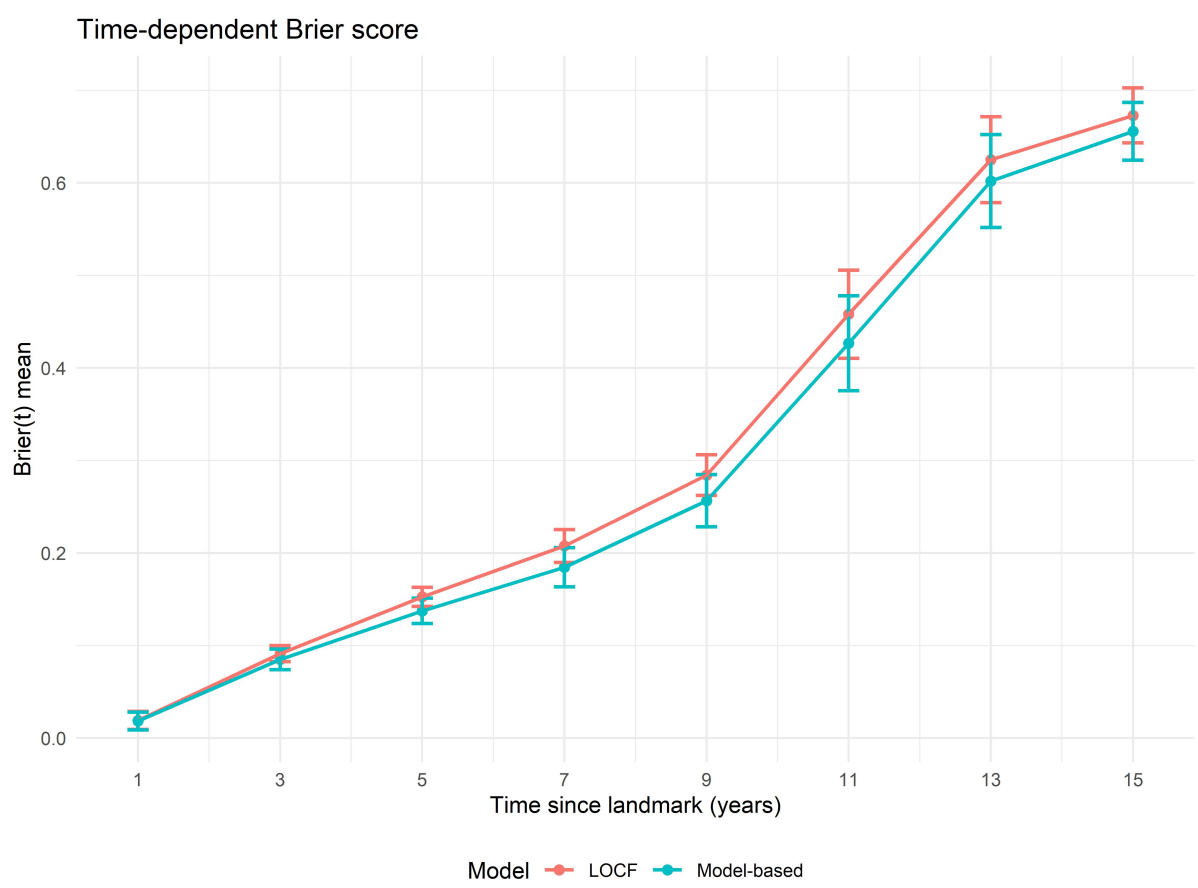

(d) Landmark time $t = 2.5$

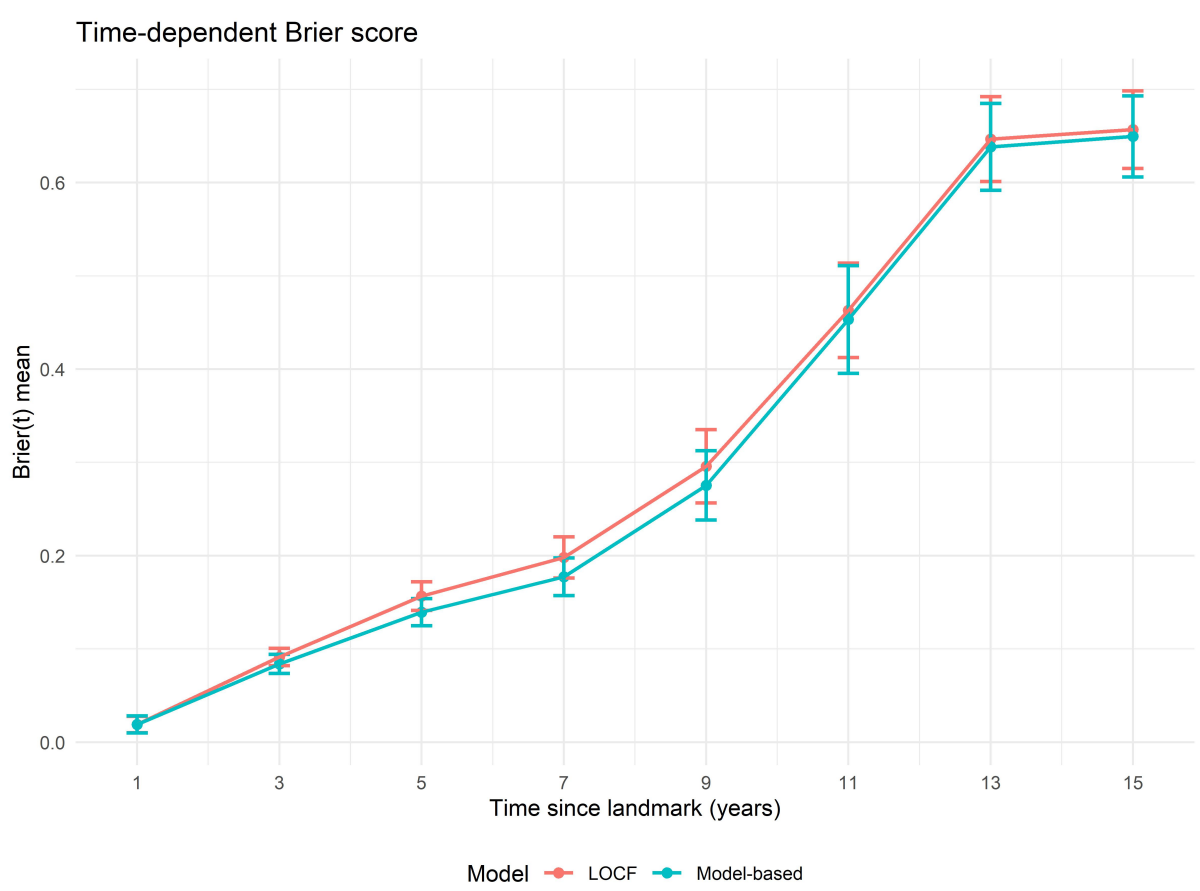

(e) Landmark time $t = 3$

Figure 7: Time-dependent Brier Score curves along with standard errors for the model-based and LOCF approaches at multiple landmark times.
